# Opposing roles of Toll-like receptor and cytosolic DNA-STING signaling pathways for *Staphylococcus aureus* cutaneous host defense

**DOI:** 10.1371/journal.ppat.1006496

**Published:** 2017-07-13

**Authors:** Philip O. Scumpia, Giovanni A. Botten, Joshua S. Norman, Kindra M. Kelly-Scumpia, Roberto Spreafico, Amber R. Ruccia, Prabhat K. Purbey, Brandon J. Thomas, Robert L. Modlin, Stephen T. Smale

**Affiliations:** 1 Department of Medicine, Division of Dermatology, University of California at Los Angeles, Los Angeles, California, United States of America; 2 Department of Microbiology, Immunology, and Molecular Genetics, University of California at Los Angeles, Los Angeles, California, United States of America; 3 Institute for Quantitative and Computational Biosciences, University of California at Los Angeles, Los Angeles, California, United States of America; Columbia University, UNITED STATES

## Abstract

Successful host defense against pathogens requires innate immune recognition of the correct pathogen associated molecular patterns (PAMPs) by pathogen recognition receptors (PRRs) to trigger the appropriate gene program tailored to the pathogen. While many PRR pathways contribute to the innate immune response to specific pathogens, the relative importance of each pathway for the complete transcriptional program elicited has not been examined in detail. Herein, we used RNA-sequencing with wildtype and mutant macrophages to delineate the innate immune pathways contributing to the early transcriptional response to *Staphylococcus aureus*, a ubiquitous microorganism that can activate a wide variety of PRRs. Unexpectedly, two PRR pathways—the Toll-like receptor (TLR) and Stimulator of Interferon Gene (STING) pathways—were identified as dominant regulators of approximately 95% of the genes that were potently induced within the first four hours of macrophage infection with live *S*. *aureus*. TLR signaling predominantly activated a pro-inflammatory program while STING signaling activated an antiviral/type I interferon response with live but not killed *S*. *aureus*. This STING response was largely dependent on the cytosolic DNA sensor cyclic guanosine-adenosine synthase (cGAS). Using a cutaneous infection model, we found that the TLR and STING pathways played opposite roles in host defense to *S*. *aureus*. TLR signaling was required for host defense, with its absence reducing interleukin (IL)-1β production and neutrophil recruitment, resulting in increased bacterial growth. In contrast, absence of STING signaling had the opposite effect, enhancing the ability to restrict the infection. These results provide novel insights into the complex interplay of innate immune signaling pathways triggered by *S*. *aureus* and uncover opposing roles of TLR and STING in cutaneous host defense to *S*. *aureus*.

## Author summary

Individual pathogen associated molecular patterns (PAMPs) induce gene expression in immune cells through distinct signaling pathways to protect cells from infection. However, pathogens typically possess many PAMPs, and the precise contribution of each PAMP to the gene expression program elicited by a live pathogen has not been clearly defined. Herein, we used gene expression profiling to examine the full early response of macrophages to *Staphylococcus aureus*, a major human opportunistic pathogen. Surprisingly, we found that two pathogen-sensing pathways, Toll-like receptor (TLR) and Stimulator of Interferon Signaling Gene (STING) pathways, contribute to the activation of ~95% of the genes induced by *S*. *aureus* infection. The remaining genes may be induced by hypoxia pathways. When the bacterium is dead, 98% of the gene induction occurs through TLR signaling, and neither STING nor hypoxia contributes greatly to the response. STING activation requires sensing of *S*. *aureus* DNA by the cytosolic DNA sensor, cGAS. During *S*. *aureus* skin infection, the TLR and STING pathways compete with each other to induce or suppress host defense, respectively, by counter regulating interleukin 1β production and neutrophil recruitment. A similar approach may allow delineation of the relative contributions of immune pathways in the response to various live pathogens.

## Introduction

Cells of the innate immune system, including macrophages and neutrophils, are tasked with initiating the rapid and robust response to invading microbial pathogens. Armed with a variety of pattern recognition receptors (PRRs), these sentinel cells sense pathogen associated molecular patterns (PAMPs) displayed by microbes to trigger the appropriate antimicrobial response. Specific gene programs activated by microbial pathogens include genes that promote inflammation, genes whose products are directly microbicidal, and genes that induce and regulate adaptive immune responses. However, pathogens may also activate a transcriptional program that interferes with host defense pathways. Understanding this complex interplay of host immune responses to pathogens and evasion of host defense by the pathogen is critical for the discovery of new therapeutics. While much work has been performed identifying how individual PAMPs trigger transcriptional cascades, less work has been performed examining how a complex, living pathogen can trigger transcriptional responses in immune cells and whether these initial transcriptional responses result in protective immunity or favor pathogen persistence.

*Staphylococcus aureus* is a Gram-positive microorganism that is the leading cause of skin and soft tissue infections in humans. Dissemination of *S*. *aureus* can result in life-threatening infections, and invasive infections with *S*. *aureus* result in more deaths annually than infections with any other infectious agent in the United States[1]. Through its acquisition of resistance to multiple antibiotics, methicillin resistant *S*. *aureus* (MRSA) has reemerged as a major public health concern. A major goal is to define the regulatory mechanisms responsible for host recognition of *S*. *aureus* while identifying pathways triggered by *S*. *aureus* to evade host defense for the purpose of developing strategies to improve immune responses and better combat infection.

Through studies of mutant mice, *S*. *aureus* has been shown to activate a myriad of PAMP/PRR pathways. Toll-like receptors (TLRs) are one such family of cell surface and endosomal PRRs that recognize a variety of microbial PAMPs. TLR1/2 or TLR2/6 recognizes cell wall components of *S*. *aureus* including Pam3CysK4 and lipotechoic acids [2,3]. RNA and DNA from *S*. *aureus* have been shown to activate TLR8 and TLR9, respectively [4,5]. Activation of TLRs results in signaling through the adaptor proteins Myeloid differentiation factor 88 (MyD88) and/or Toll-IL-1 receptor domain containing adaptor inducing interferon-β (TRIF) to initiate transcriptional responses. Additionally, *S*. *aureus* can either escape endosomes to enter the cytosol [6,7] or secrete various virulence factors into the cytosol from within the endosome, including cell wall components (muramyl dipeptide), toxins, secreted substances, or DNA; these factors can trigger cytosolic receptors, such as nucleotide oligomerization domain (NOD) 2, the NOD-like receptor (NLR) NLRP3, and Absent in melanoma 2 (AIM2), respectively. Many of the above pathways converge to activate IL-1β transcription (via TLRs) and processing (via the inflammasome), with IL-1β serving as a critical host defense factor required to control *S*. *aureus* [8–10].

While the above pathways are involved in host defense, *S*. *aureus* can also induce type I interferon (IFN) production [11], which has been associated with evasion of host defense. This type I IFN has been shown to directly inhibit IL-1β transcription and IL-1β processing through the inflammasome in response to multiple infections [12–14], decreasing host defense in certain infections, but diminishing systemic inflammation in response to others [15]. A variety of pathways have been reported to contribute to the type I IFN response, including the aforementioned TLR2 [16], TLR8 [5], TLR9 [4], and NOD2 pathways [17], as well as a recently identified pathway involving activation of Stimulator of IFN Genes (STING) [18]. While STING activation was shown to be dependent upon DacA-mediated production of the small molecule, di-cyclic AMP in response to extracellular *S*. *aureus* biofilms, a cytosolic DNA sensor, cyclic GMP-AMP synthase (cGAS), can also activate STING in response to cytosolic DNA from many other bacterial pathogens [19–22].

TLR pathways can be triggered by ligands present on either living or killed pathogens taken up from the extracellular milieu, whereas the cytosolic PRR families only sense living pathogens that can gain access to the cytosol (through direct invasion or secretion of factors). Although DNA microarray analyses of mRNA from infected macrophages have allowed an examination of individual host defense pathways in the response to pathogens such as *Mycobacterium tuberculosis* and *Listeria monocytogenes* [23–25], a comprehensive analysis of the relative contributions of multiple PRR pathways has not been performed. Furthermore, how these pathways enhance or inhibit early host defense has not been fully elucidated.

Herein, we defined the transcriptional programs induced by live and heat-killed (HK) *S*. *aureus* using RNA Sequencing (RNA-seq) of macrophages from wild type (WT) and mutant mice. We examined the extent to which each of several pathways contributes to the *S*. *aureus* response. Live and HK *S*. *aureus* induced similar transcriptional programs, but utilized different sensing mechanisms for the induction of many genes. While TLR signaling pathways were responsible for a high percentage of the response to HK *S*. *aureus*, live *S*. *aureus* utilized both TLR and STING signaling to induce the transcriptional program. The STING response required live bacteria, was activated by multiple Staphylococcal species, and was activated in mouse and human cells predominantly through cGAS recognition of *S*. *aureus* DNA. Interestingly, a small number of TLR- and STING-independent genes activated by live *S*. *aureus* were linked to a hypoxia sensing pathway. Using a cutaneous *S*. *aureus* infection model, we found that, while activation of TLRs through MyD88 resulted in protective immunity by inducing IL-1β production, neutrophil infiltration, and neutrophil activity in the skin, activation of STING antagonized protective immunity by limiting neutrophil recruitment and IL-1β production. These findings highlight the complex interplay between TLR-dependent host defense and cGAS-STING-dependent immune evasion mechanisms in the early transcriptional responses elicited by *S*. *aureus*.

## Results

### Live *S*. *aureus* induces a rapid and robust gene expression program in cultured macrophages

To fully understand the gene programs induced by infection with live *S*. *aureus*, we first used RNA-seq to characterize the genome-wide response to the bacterium in WT mouse C57BL/6 bone marrow-derived macrophages (BMDMs). During initial experiments, we found that the rapid proliferation of live *S*. *aureus* can quickly overtake BMDM cultures. We therefore examined the gene expression program during the first four hours of culture, prior to bacterial overgrowth. We did not add antibiotics to the culture medium as this would skew responses to those induced by killed bacteria. We also found that cultured BMDMs were exquisitely sensitive to hemolysin-producing *S*. *aureus* strains, which induced considerable cell death (30–40%) within two hours. To focus on immune pathways and minimize the effects of apoptosis and cell stress pathways on the transcriptional signature, we used the DU5938 (α, β, γ hemolysin-deficient) strain of *S*. *aureus* [26], which induced only minimal cell death (~10%) during the four-hour culture period.

We first examined the gene expression profile of mouse BMDMs cultured with live *S*. *aureus* (MOI 10). We focused on highly expressed genes (at least 1 RPKM at one time point) that were strongly and significantly induced (at least 5-fold at one or more time points, at p-adjusted<0.05 by DESeq) for all subsequent analyses. Inclusion of genes induced more weakly would have made the results of loss-of-function experiments more difficult to interpret. 369 genes met our induction criteria, and we focused the remainder of our analysis on these genes.

**Fig 1:**
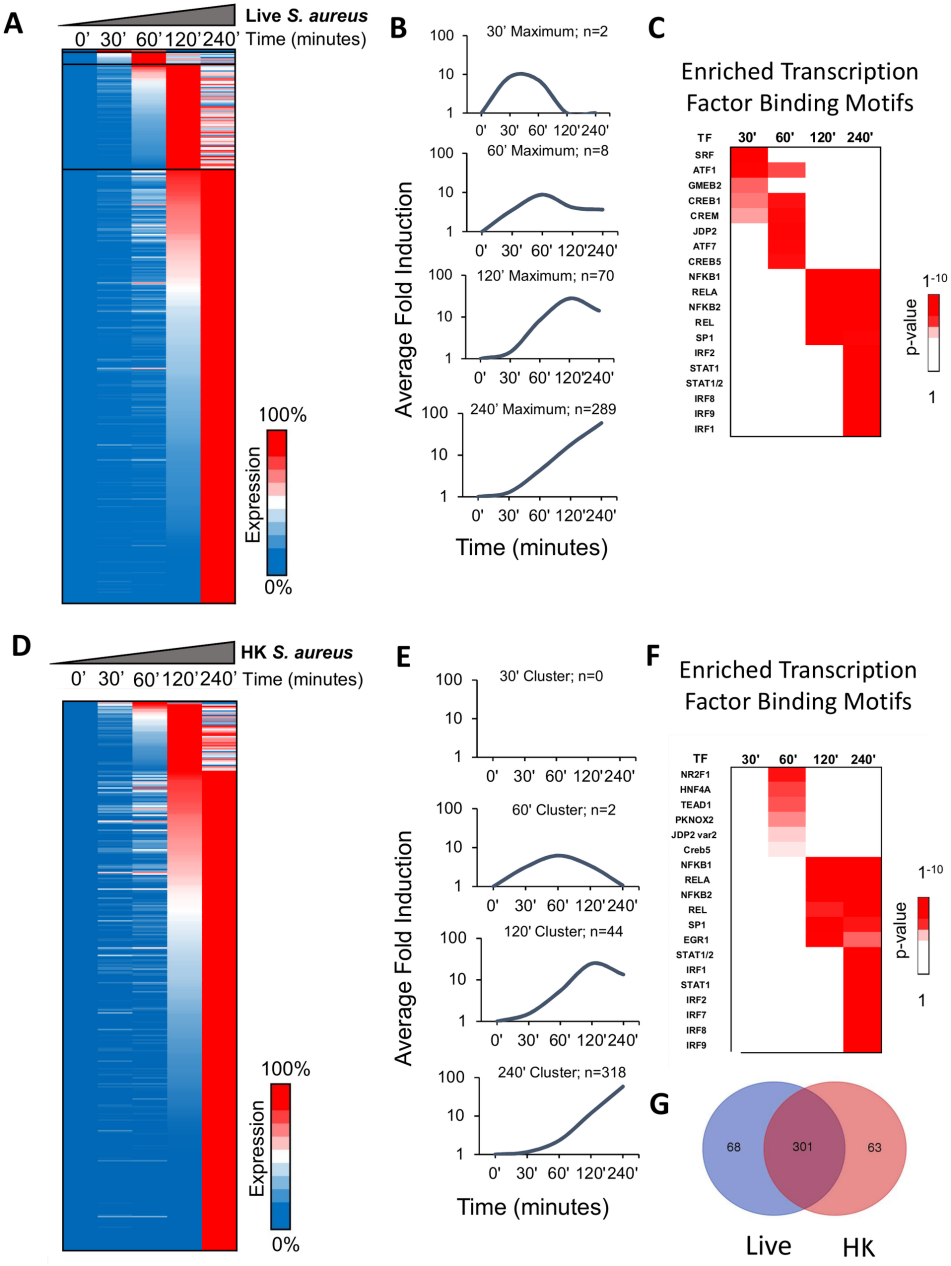
Kinetic properties of the transcriptional cascade to live and HK *S*. *aureus*. (A) Activation kinetics are shown for inducible genes from BMDMs stimulated with an MOI of 10 of *S*. *aureus* at indicated time points. Shades of blue to red indicate percentile values from low to high expression. Shown are all genes meeting RPKM >1, Fold change >5, and p<0.05 by DESeq. The average of at least 4 separate experiments is shown for each time point (B) The average fold of transcript levels of inducible genes separated within representative clusters by time point the genes reach their maximal expression level are shown. (C) Known, highly significant Jaspar 2016 motif enrichment of genes which reach maximal expression levels at the indicated time points following *S*. *aureus* infection. (D) Activation kinetics are shown for inducible genes from BMDMs stimulated with the equivalent of an MOI of 10 HK *S*. *aureus* at indicated time points. Shades of blue to red indicate percentile values from low to high expression. Induction and expression criteria are as in A. Shown are all genes meeting RPKM >1, Fold change >5, and p<0.05 by DESeq. The average of at least 2 separate experiments is shown for each time point (E) The average fold of transcript levels of inducible genes separated within representative clusters by time point the genes reach their maximal expression level are shown. (F) Known, highly significant Jaspar 2016 motif enrichment of genes which reach maximal expression levels at the indicated time points following *S*. *aureus* infection. (G) Venn diagram showing degree of overlap between all genes which meet expression and induction threshold criteria in macrophages in response to live or HK *S*. *aureus* treatment.

We next separated the 369 genes into four clusters based on the time point of maximum expression (Fig 1A and 1B). In general, genes that reached their maximum expression at later time points were more strongly induced than genes that peaked early in the time course (Fig 1B). Only two genes, *Fos* and *Egr1*, reached peak expression within 30 minutes of treatment with live *S*. *aureus* (Fig 1A). These genes are well-known targets of serum response factor (SRF), and their promoters displayed enrichment of binding sites for SRF, Activating Transcription Factor (ATF), and cyclic AMP Response Element Binding (CREB) (Fig 1B and 1C). Motifs for additional transcription factors were enriched in this analysis and in those shown below, but we will focus on those that have been studied most extensively.

Genes that reached peak expression at 60 minutes also were induced by small magnitudes (10-fold average). However, unlike the transiently induced genes that reached maximal expression at 30 minutes, these genes generally maintained substantial expression throughout the remainder of the four-hour time course (Fig 1B). These genes also displayed enrichment for ATF and CREB binding sites in their promoters, but lacked significant SRF site enrichment (Fig 1C).

The largest groups of genes induced by live *S*. *aureus* reached their peak expression at 120 minutes or 240 minutes. The 70 genes induced maximally at 120 minutes displayed a greater range of induction (5- to 313-fold) and, on average, were induced more strongly (27-fold) than those induced maximally at earlier time points. The promoters of these genes possessed significant enrichment for NF-κB family members binding sites, consistent with classic inflammatory genes (Fig 1B and 1C) [27].

The average fold induction for the 289 genes that peaked at 240 minutes was considerably higher (57-fold average, range 5- to 1617-fold). The promoters of these genes also exhibited enrichment for NF-κB family member binding sites, but additionally exhibited enrichment for IFN Regulatory Factor (IRF) and Signal Transducer and Activator of Transcription (STAT)1 and STAT2 binding sites; these factors are often associated with a type I IFN response (Fig 1B). Overall, these findings highlight a highly ordered transcriptional response, with different sets of transcription factors coordinating the transient and sustained responses to live *S*. *aureus*, similar to previous findings with macrophages treated with a TLR4 agonist [27,28].

### Live and HK *S*. *aureus* induce similar gene expression responses

We next compared the macrophage gene expression responses to live and HK *S*. *aureus*. RNA-seq was performed with BMDMs treated with HK *S*. *aureus* (equivalent to MOI = 10) and the data sets were analyzed as above. We found 364 genes that met the induction criteria (>5-fold induction; maximum expression >1 RPKM; p-adjusted<0.05) following treatment with HK *S*. *aureus*, similar to the number induced by live *S*. *aureus* (369) (Fig 1D and 1E). The kinetics of the response to HK *S*. *aureus* was similar to, but slightly slower than, the response to live *S*. *aureus* (Fig 1D and 1E).

Enrichment of transcription factor binding motifs was similar to that observed with live *S*. *aureus*, with CREB motifs associated with the promoters of early genes, followed by NF-κB motifs, and finally NF-κB, STAT and IRF motifs enriched in the promoters of genes that peaked at the 240-minute time point (Fig 1F). *Fos* and *Egr1*, the two genes significantly induced at 30 minutes by live *S*. *aureus*, were weakly induced (between 3-4-fold) at the 30-minute time point, but they did not meet the 5-fold or p-adjusted <0.05 criteria and therefore were excluded from the analysis (S1 Table).

In total, our gene sets include 432 genes that were induced by either live or HK *S*. *aureus*, or both (Fig 1G). Although 68 genes were considered to be unique to the live *S*. *aureus* set and 63 genes were considered to be unique to the HK *S*. *aureus* set, the vast majority of these genes were actually induced by both the live and HK organism; they simply missed the thresholds required for inclusion in one of the gene sets (S1A and S1B Fig), with most missing the 5-fold induction threshold with the second stimulus. Of the 63 genes appearing uniquely induced by HK *S*. *aureus*, 57 were, in fact, induced 3-5-fold by live *S*. *aureus*, with only 6 genes being induced 2–3 fold (S1B Fig). Similarly, most of the 68 genes that appeared uniquely induced by live *S*. *aureus* were also induced 3–5 fold by HK *S*. *aureus*; however, 20 genes did not reach 3-fold induction, and 9 of these genes were not induced 2-fold (S1B Fig).

These experiments revealed a highly similar overall progression of the transcriptional responses induced by live and HK *S*. *aureus*, with the exception of a very small cluster of genes induced by live but not HK *S*. *aureus*. The findings therefore support an initial hypothesis that similar PRR pathways are responsible for gene induction by the live and HK organisms.

### Defining the role of TLR signaling in the overall gene expression response to live *S*. *aureus* in macrophages

Our next goal was to provide a mechanistic framework for gene induction by *S*. *aureus*. Because *S*. *aureus* possesses agonists to various TLRs, we first examined the effect of abolishing all TLR-dependent signaling through an analysis of BMDMs from *Myd88*^*-/-*^*Trif*^*-/-*^ mice; all TLR-dependent signaling is thought to be abolished in these mice because all TLRs are thought to use MyD88 and/or TRIF as essential downstream adaptors.

**Fig 2:**
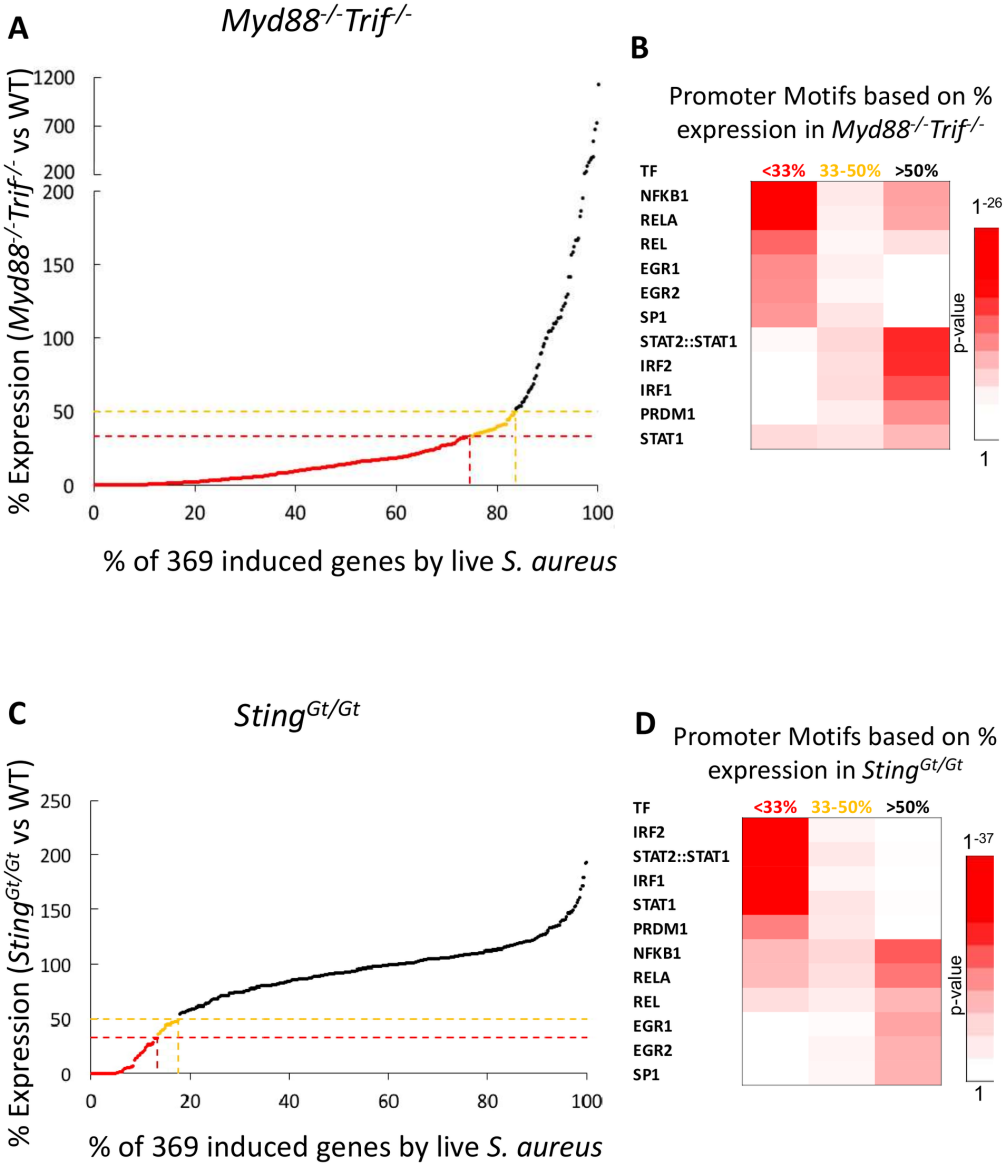
Toll-like receptor and STING signaling dominate the transcriptional response to *S*. *aureus* in murine macrophages. (A) RNA-Seq of *Myd88/Trif*^*-/-*^ macrophages was compared to that of WT macrophages. Shown is the % expression value of the 369 genes induced by live *S*. *aureus* in *Myd88/Trif*^*-/-*^ macrophages compared to WT macrophages. The average expression from two separate experiments of WT and *Myd88/Trif*^*-/-*^ macrophages treated at the same time with the same dose of *S*. *aureus* at 5 different time points in each experiment is shown. (B) Known, highly significant Jaspar 2016 motif enrichment of genes separated by their degree of dependence on *Myd88/Trif*^*-/-*^ following *S*. *aureus* infection. (C) RNA-Seq of *Sting*^*Gt/Gt*^ macrophages was compared to that of WT macrophages. Shown is the % expression value of the 369 genes induced by live *S*. *aureus* in *Sting*^*Gt/Gt*^ macrophages compared to WT macrophages. The average expression from two separate experiments of WT and *Sting*^*Gt/Gt*^ macrophages treated at the same time with the same dose of *S*. *aureus* at 5 different time points in each experiment is shown. (D) Known, highly significant Jaspar 2016 motif enrichment of genes separated by their degree of dependence on STING following *S*. *aureus* infection.

RNA-seq was performed in parallel with mRNA from WT and *Myd88*^*-/-*^*Trif*^*-/-*^ BMDMs treated with live *S*. *aureus* for 0, 30, 60, 120, and 240 minutes. Our initial analysis revealed that 308 of the 369 genes induced by live *S*. *aureus* in WT mice were expressed in the mutant cells at a level that was less than 50% of the WT level; 276 of those genes were expressed at a level that was less than 33% of the WT level (<33% expression; Fig 2A). Motifs for NF-κB family members were most strongly enriched in the promoters of the genes that exhibited the greatest MyD88/TRIF-dependence (Fig 2B). In contrast, motifs for IRF and STAT family members (and NF-κB to a lesser extent) were most strongly enriched in the promoters of the genes that exhibited little or no dependence on MyD88/TRIF (Fig 2B). These results confirm a major role for TLR/MyD88/TRIF signaling and NF-κB in the early inflammatory response activated by live *S*. *aureus*, with an apparent association between TLR/MyD88/TRIF-independent genes and IRF/STAT factors that may be reflective of a type I IFN response.

### A broader role for TLR/MyD88/TRIF signaling in the response to HK *S*. *aureus*

We next performed RNA-seq with mRNA from WT and *Myd88*^*-/-*^*Trif*^*-/-*^ BMDMs macrophages treated with HK *S*. *aureus* for 0, 30, 60, 120, and 240 minutes. Interestingly, in response to HK *S*. *aureus*, the induction of a robust TLR-independent program was absent; even the type I IFN program reflected in the enrichment of STAT/IRF motifs was found to be dependent on TLR signaling in response to HK *S*. *aureus* (S2A and S2B Fig). In fact, only 6 of the 364 genes induced by HK *S*. *aureus* were induced to 50% of WT values, confirming that TLRs are the dominant PRRs that contribute to nearly the entire early transcriptional program in response to HK *S*. *aureus*, including the type I IFN signature (S2A–S2C Fig). These data also suggest that while TLR signaling is fully capable of inducing a type I IFN response in macrophages treated with dead *S*. *aureus*, macrophages utilize a different mechanism to induce a type I IFN dependent program in response to live *S*. *aureus*.

### Identification of STING as the mediator of the type I IFN program in response to live *S*. *aureus*

Many immune pathways have been reported to contribute to the induction of the type I IFN response by live *S*. *aureus*, but several of these pathways would be inactive in *Myd88*^*-/-*^*Trif*^*-/-*^ macrophages [4,5,11,16]. Only a few MyD88/TRIF-independent PRR pathways are triggered by living microorganisms and capable of inducing type I IFN, including the NOD2, STING, and retinoic acid-inducible gene I (RIG-I)/mitochondrial antiviral signaling (MAVS) pathways. While a NOD2-IRF5 pathway was shown to induce type I IFN in epithelial and dendritic cells in response to a different strain of *S*. *aureus* (502A), the *S*. *aureus* strain more likely to cause disease, USA300, was a poor activator of this pathway [17]. Consistent with these earlier findings, in preliminary qRT-PCR studies, we were unable to detect a role of NOD2 in the response to live *S*. *aureus* at select TLR signaling-dependent (*Tnf*, *Il6*, *Cxcl1*) and -independent (*Rsad2*, *Ifit1*, *Ifit3*, *Mx1*, *Mx2*) genes. Similarly, *Mavs*^*-/-*^ macrophages did not reveal diminished expression of the above genes tested in response to live *S*. *aureus*, consistent with previous reports demonstrating no role of MAVS in *S*. *aureus*-induced type I IFN production [29]. We next tested whether STING is responsible for the type I IFN response in macrophages treated with live *S*. *aureus*.

In preliminary experiments, BMDM from *Sting*^*Gt/Gt*^ mutant mice [30] demonstrated an impairment in the induction of several type I IFN-induced genes in response to live *S*. *aureus* when compared to WT BMDMs. We therefore used RNA-seq to compare the transcriptional programs in these two strains. We found that 65 genes induced by live *S*. *aureus* were expressed at a level that was <50% of the WT expression level, and 49 of these genes were expressed at a level that was <33% of the WT expression level, in *Sting*^*Gt/Gt*^ macrophages (Fig 2C). Promoter enrichment analysis revealed strong IRF and STAT signatures in the promoters of genes regulated strongly by STING (Fig 2D). The genes that reached >50% of WT expression level in the absence of STING signaling possessed strong NF-κB and EGR1/EGR2 signatures. Genes that only reached 33–50% of WT expression level in mice devoid of STING signaling displayed modest promoter enrichment for IRF/STAT, NF-κB, and EGR binding sites, again suggesting that both TLR- and STING- dependent signals can regulate the induction of these partially inhibited genes (Fig 2D).

We next examined whether STING participates in the early transcriptional response to HK *S*. *aureus*. We found that the vast majority of genes that were induced by HK *S*. *aureus* in WT macrophages were induced similarly in STING^Gt/Gt^ macrophages (S1A and S1B Fig). Only 14 genes showed <50% of WT expression, and only 1 of those genes displayed <33% of WT expression, in *Sting*^*Gt/Gt*^ macrophages. All of these genes were also inhibited in *Myd88*^*-/-*^*Trif*^*-/-*^ macrophages to a similar or greater degree, and they did not display a strong IRF or STAT signature; they instead displayed POU2F1 and RUNX2 signatures and a weaker NFκB1 signature, highlighting a very minor, if any, role for STING in the response to HK *S*. *aureus*.

### Building a framework of PRR pathways responsible for the live *S*. *aureus*-induced transcriptional program

Upon further analysis, while the majority of the genes that displayed impaired expression in *Sting*^*Gt/Gt*^ macrophages did not display similar impairment in *Myd88*^*-/-*^*/Trif*^*-/-*^ macrophages, several genes displayed partial inhibition in the absence of either pathway. We therefore wished to analyze the datasets simultaneously to obtain an overall framework of the role of TLR and STING dependent signaling to the response to live *S*. *aureus*.

**Fig 3:**
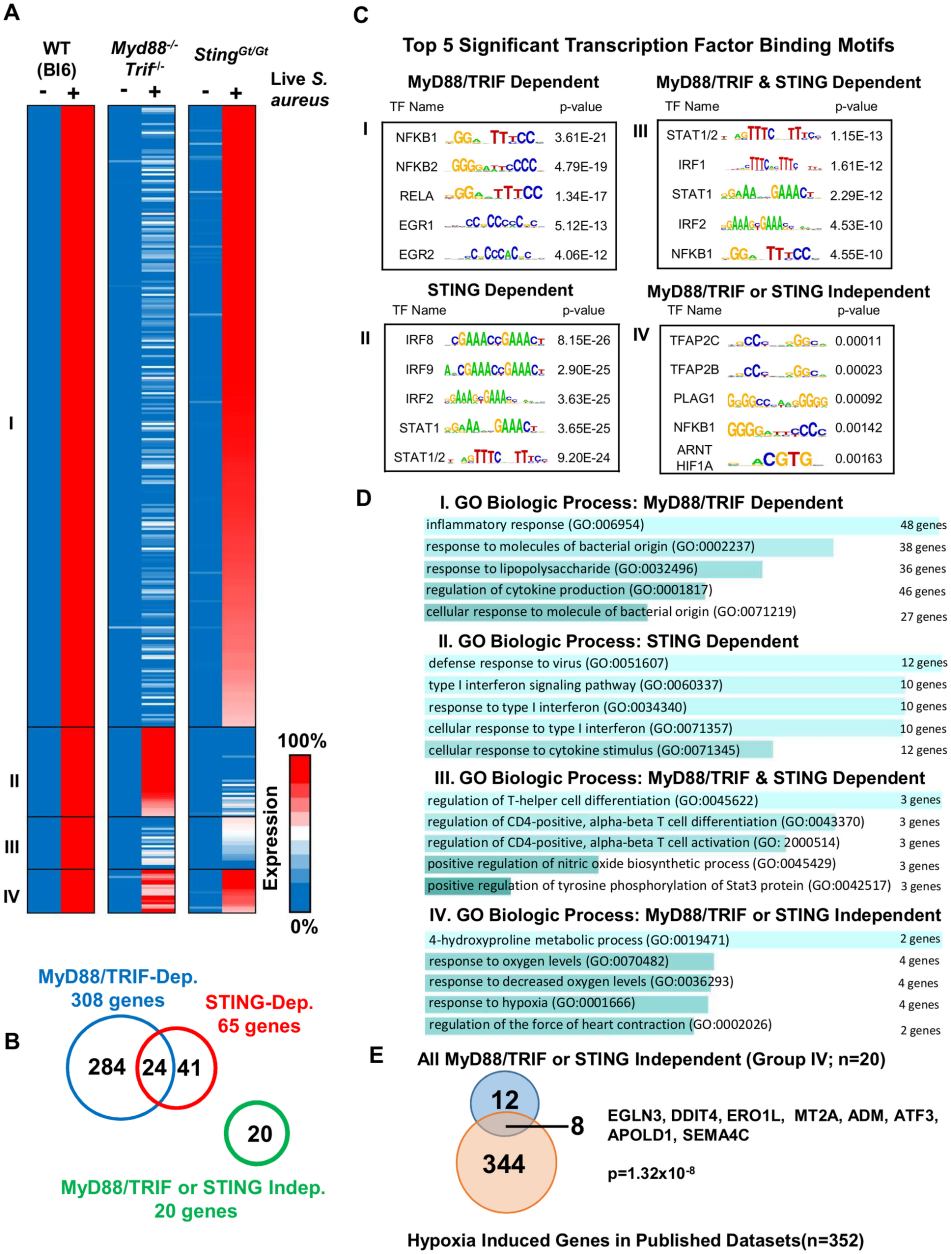
Integrated framework of transcriptional activation of genes induced in BMDMs by live *S*. *aureus*. (A) Heat map of percentile induction of genes induced by treatment of BMDMs with live *S*. *aureus* (MOI 10) in wild type (B6), *Myd88*^*-/-*^*Trif*^*-/-*^, or *Sting*^*Gt/Gt*^ mice reveals four distinct clusters of genes. Genes are separated into 4 clusters (I, II, III, and IV) based on mode of induction. (B) Venn diagram demonstrating the breakdown of genes in the four clusters of genes based on >50% dependence on the two main pathways induced. (C) Enriched Jaspar 2016 motifs within promoters of genes within the 4 clusters of genes defined by dependence on TLR and/or STING pathways. (D) GO Biologic Process reveals functions of genes within each cluster. The number of genes listed next to the GO Biologic Process number refers to the number of *S*. *aureus*-induced genes in our dataset that contribute to the significant enrichment of the GO term dataset. (E) Comparison of “MyD88/TRIF or STING Independent” cluster of 20 genes with known monocyte/macrophage datasets stimulated with hypoxia reveals significant overlap of signatures (p-value by Fisher's Exact Test).

We identified 4 groups of genes based solely on their need for TLR and/or STING signaling for >50% expression. The majority of live *S*. *aureus*-induced genes (284/369) was diminished to <50% of WT expression in the absence of TLR signaling (Group I; Fig 3A and 3B). The transcription factor binding motifs in the promoters of these genes, not surprisingly, exhibited strong enrichment for NF-κB motifs (Fig 3C; S1 Table). When Gene Ontology (GO) Biologic Process analysis [31] was used to evaluate this gene family, the main processes included inflammatory response, response to molecules of bacterial origin or lipopolysaccharide, and regulation of cytokine production (Fig 3D and S3A Fig). These results highlight the strong role of TLR signaling in the induction of the early inflammatory response induced by live *S*. *aureus*.

The next transcriptional program included a group of 41 genes whose induction was diminished to <50% of WT in the absence of STING signaling (Group II; Fig 3A and 3B; S1 Table). The promoters of these genes were enriched in IRF and STAT motifs, which are associated with a type I IFN response (Fig 3C). GO Biologic Process analysis confirmed that this group exhibited an enrichment of genes associated with a type I IFN gene program; the enriched processes included defense response to virus, type I IFN signaling pathway, response to type I IFN, cellular response to type I IFN, and cellular response to cytokine stimulus (Fig 3D and S3A Fig).

The third group includes 24 genes induced by live *S*. *aureus* that was diminished to <50% of WT in macrophages devoid of either TLR- and STING-signaling (Group III; Fig 3A and 3B; S1 Table). These genes display strong enrichment for STAT1/2, IRF1, IRF2, STAT1 and NFκB1 promoter motifs (Fig 3C). When GO Biologic Process analysis was applied to this group, enrichment was observed for regulation of T-helper cell differentiation, regulation of CD4-positive alpha-beta T cell differentiation and activation, positive regulation of nitric oxide biosynthetic processes, and tyrosine phosphorylation of STAT3 protein (Fig 3D and S3A Fig). Not surprisingly, a subset of these genes, including *Il12b*, *Il6*, *Il27*, and *Nos2*, are among the most strongly induced genes by PAMPs in macrophages, and both the TLR and type I IFN programs can result in their induction. These results suggest that Group III genes require strong and/or multiple signals for their maximal induction [27].

The final group of 20 genes was induced >50% of WT in the absence of either TLR- or STING dependent signals (Group IV; Fig 3A and 3B; S1 Table). Interestingly, promoter motif analysis revealed weak enrichment for hypoxia inducible factor (HIF)-1α motifs (Fig 3C). When GO Biologic Process analysis was applied, 4-hydroxyproline metabolism, response to oxygen levels, response to decreased oxygen levels, and response to hypoxia were the major biological processes found to be enriched (Fig 3D and S3A Fig), suggesting that treatment with live *S*. *aureus* induced a hypoxia response or activated HIF-1 dependent signaling independently of TLR and STING signaling.

### A hypoxia signature is enriched in the absence of TLR or STING signaling

To gain further insight into whether a hypoxia response was in fact induced by live *S*. *aureus*, we tested whether this group of genes had been previously found in hypoxic macrophages. Indeed, previous work suggested that a metabolic/bioenergetic phenomenon occurs upon infection of cells or tissues with live pathogens, including *S*. *aureus*, resulting in the PRR-independent induction of a hypoxic response [32]. We compiled a list from 4 manuscripts [33–36] that tested the direct effects of hypoxia on human or mouse myeloid populations in culture. While undoubtedly TLR signaling can trigger a hypoxia response [37], we focused only on those genes that were induced strongly in the absence of TLR- or STING-dependent signaling, as it appeared that *S*. *aureus* was inducing an additional hypoxia response that did not require TLR signaling. When the 20 genes that did not display an appreciable TLR/STING requirement in our analysis were compared against a list of 352 genes derived from 4 datasets of hypoxia-induced genes in monocytes or macrophages, 8 of our 20 genes were found in the hypoxia-induced gene set (Fig 3E; p = 1.32e-08).

Next, we examined whether HK *S*. *aureus* induced the genes associated with the hypoxia signature. We created a similar framework using *Myd88*^*-/-*^*Trif*^*-/-*^ and *Sting*^*Gt/Gt*^ macrophages treated with HK *S*. *aureus*. We found only 3 groups of genes based on TLR and/or STING signaling (S1A and S1B Fig). These include a group of 340 genes diminished to <50% of WT in the absence of TLR signaling, 18 genes that were diminished to <50% of WT when either TLR and STING pathways were inactivated, and 6 genes that induced at >50% of WT expression levels in the absence of either TLR or STING signaling (Groups I, II, and III, respectively, S1A Fig). Notably, all 18 genes that were diminished to <50% of WT in the absence of STING were diminished to a similar or greater extent in the absence of TLR signaling.

Of note, the promoters of Group I genes were enriched for NF-κB, IRF, and STAT motifs and included many of the genes that were diminished to <50% of WT in the absence of STING signaling or in the absence of either TLR and STING signaling in response to live *S*. *aureus* (S1C Fig, S1 and S2 Tables). The Group II genes in response to HK *S*. *aureus* exhibited promoter enrichment for IRF and STAT signatures, while the Group III genes did not have significant enrichment for any transcription factor binding motifs, as it is often difficult to obtain motif enrichment with only 6 genes (S1 Fig). Interestingly, similar to the effects of live versus HK group A Streptococcus on cultured macrophages [21], HK *S*. *aureus* was a more potent inducer of many of the genes that exhibited STING-dependence in response to live *S*. *aureus*, including *Ifnb*, *Cxcl9*, *Cxcl10*, *Il12b*, *Il27*, *Ifit1*, *Oasl1*, *and ligp1*; this finding suggests that HK *S*. *aureus* activates the type I IFN response through TLRs more strongly than live *S*. *aureus* does through STING (S1 and S2 Tables).

**Fig 4:**
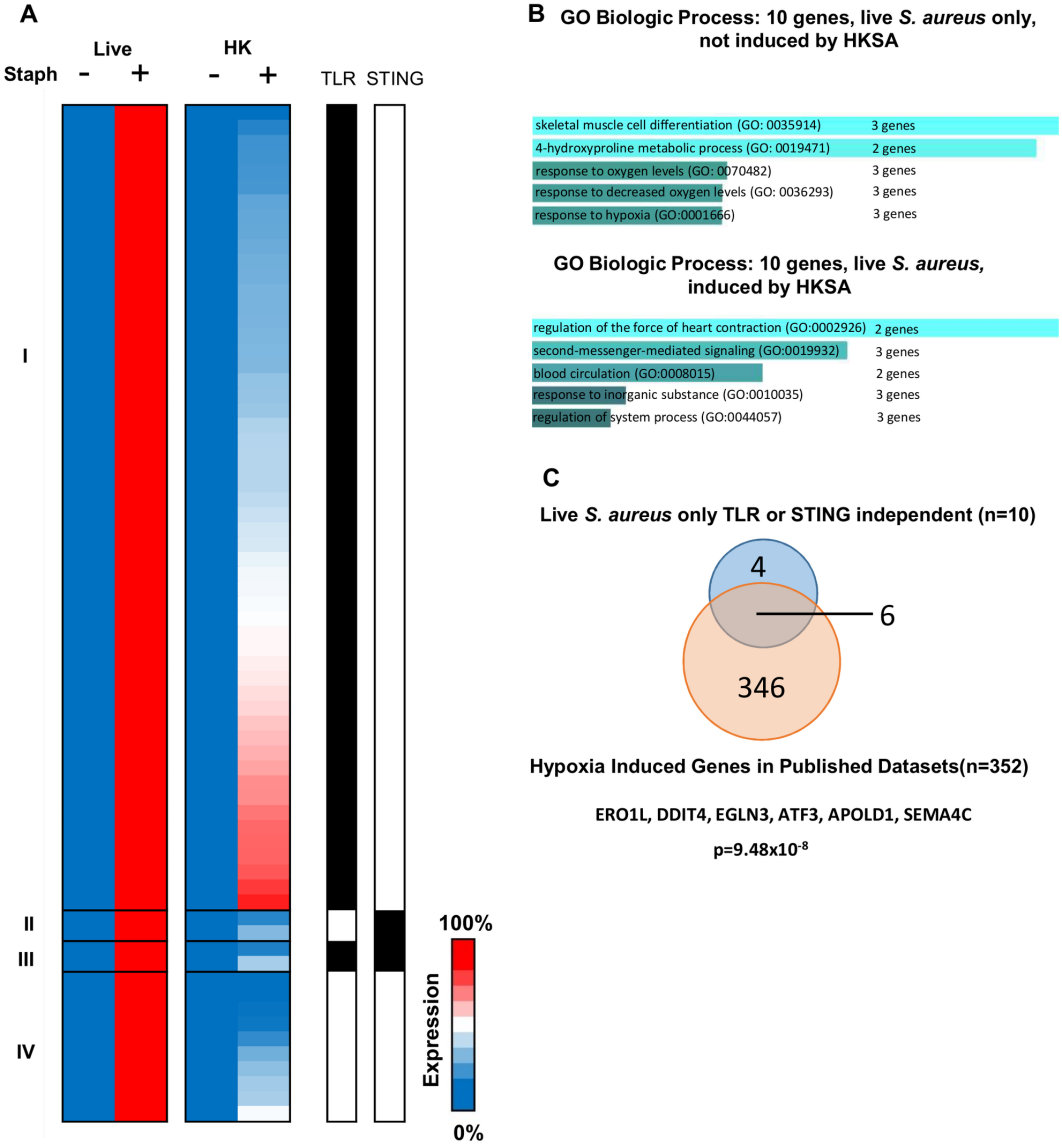
Modes of regulation of a gene signature induced by live *S*. *aureus* but not HK *S*. *aureus*. (A) Heat map demonstrating 68 genes were induced by live *S*. *aureus* that were not significantly induced by HK *S*. *aureus* (failed RPKM, fold change, or significance criteria) in BMDMs. TLR signaling was required for the induction of the majority of these genes induced by live *S*. *aureus* but not HK *S*. *aureus*, with only 2 requiring STING, and 2 genes requiring both TLRs and STING. 11 genes were induced independently of MyD88/TRIF or STING signaling in response to live *S*. *aureus* that failed to be induced by HK *S*. *aureus*. (B) GO Biologic Process of the 20 genes separated by whether they were (10 genes) or were not (10 genes) also induced by HK *S*. *aureus*, reveals genes induced by live bacteria possess the hypoxia signature. The number of genes listed next to the GO Process number refers to the number of *S*. *aureus*-induced genes in our dataset that contribute to the significant enrichment of the GO term dataset. (C) Comparison of the 10 “MyD88/TRIF or STING Independent” cluster of 20 genes that are not induced by HKSA with known monocyte/macrophage datasets stimulated with hypoxia reveals significant overlap of signatures (p-value by Fisher Exact Test).

To evaluate whether the hypoxia program required the presence of live *S*. *aureus*, we further examined the 68 genes that were strongly induced by live but not by HK *S*. *aureus* (see Fig 1G). As mentioned above, several of these genes were induced by HK *S*. *aureus*, but did not reach the thresholds needed for inclusion in our gene sets. Surprisingly, 54 of these 68 genes were diminished to <50% of WT in the absence of TLR signaling (Fig 4A, Group I), with only 2 genes each exhibiting the same degree of impairment in the absence of either STING signaling alone or either TLR or STING signaling (Fig 4A, Groups II and III). The remaining 10 genes were induced when either TLR or STING pathways were not functional (Fig 4A, Group IV). These genes included 10 of the 20 genes found to be induced in the absence of TLR- or STING-signaling in response to live *S*. *aureus* (Group IV from Fig 3).

When GO Biologic Process analysis was applied to the 10 genes that are induced by live but not HK *S*. *aureus* (Fig 4A Group IV genes), response to oxygen levels, response to decreased oxygen levels, response to hypoxia, and 4-hydroxyproline metabolism were found to be the most strongly enriched GO signatures (Fig 4B and S3B Fig) and included 6 of the 8 genes that overlapped with published datasets of hypoxia stimulated macrophages (Fig 4C). These results therefore provide strong confirmation that the hypoxia response is induced specifically by live *S*. *aureus*, and that this response accounts for most of the TLR/STING-independent genes. Notably, the 10 remaining genes from the group of 20 that were induced by live *S*. *aureus* in a TLR/STING-independent manner (Fig 3, Group IV) exhibited enrichment for second messenger-mediated signaling and cardiac contractility when examined by GO Biologic Process analysis, but lacked enrichment for hypoxia (Fig 4B).

### Clinically relevant *S*. *aureus* strains activate the cGAS-STING pathway in mouse and human immune cells

**Fig 5:**
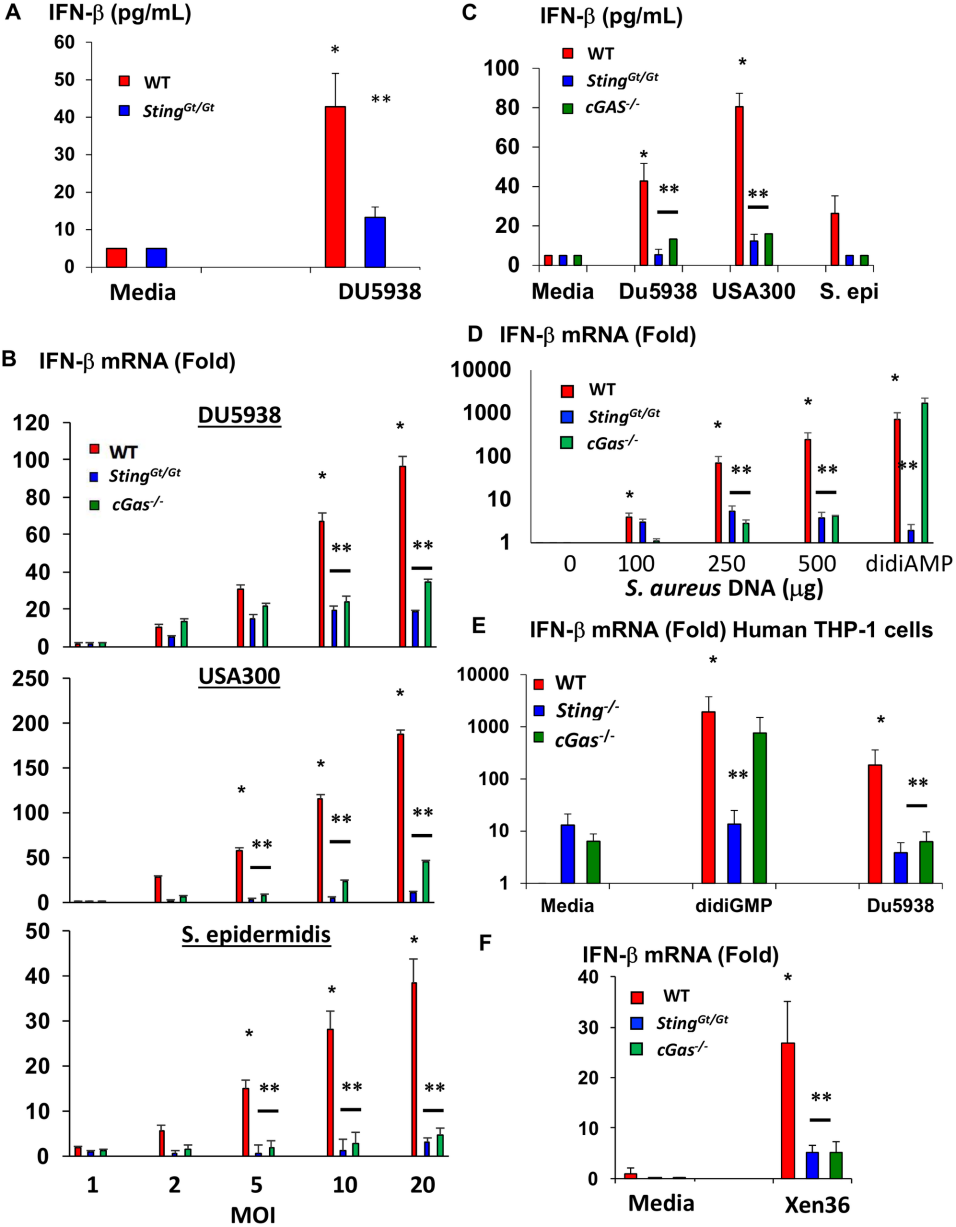
Multiple strains of staphylococcus induce IFN-β in mouse and human cells through the cGAS-STING pathway. (A) IFN-β production by BMDMs generated from WT and *Sting*^*Gt/Gt*^ mice after 4 hours stimulation with MOI 10 of strain DU5938 of *S*. *aureus*. Data shown is from individual samples from 3 separate experiments. (B) qPCR of mouse WT, *Sting*^*Gt/Gt*^, and *cGAS*^*-/-*^ BMDM stimulated with increasing MOI of the indicated species of staphylococcal organism. Experiments were performed in triplicate, and representative of two separate experiments. (C) IFN-β production by BMDMs generated from WT, *Sting*^*Gt/Gt*^,and *cGas*^*-/-*^ mice following a 4 hour treatment with an MOI 10 of DU5938 or a USA300 (JE2) strains of *S*. *aureus* (n = 3), or the same MOI of *S*. *epidermidis* (n = 2). (D) IFN-β qPCR following treatment of mouse macrophages with highly purified DNA from USA300 (JE2) strain of *S*. *aureus* grown overnight introduced intracellularly with lipofectamine for 4 hours or treated with 10 mM of the STING activator di-cyclic AMP (didiAMP). (E) IFN-β qPCR following 4 hour treatment of WT human THP-1 cells or THP-1 cells with CRISPR/Cas9 mediated deletion of STING or cGAS with di-cyclic GMP (1ug/ml) or an MOI 10 of DU5938 of S. aureus for 4 hours. (F) IFN-β qPCR following 4-hour treatment of mouse BMDMs with an MOI of 10 of the bioluminescent Xen36 *S*. *aureus* strain. For all above * denotes p<0.05 by one way ANOVA with Tukey test compared to baseline control. ** denotes p<0.05 from WT stimulated macrophages at the same time point with the same stimulation by one way ANOVA with Tukey test. All error bars denote standard error of the mean.

Because live *S*. *aureus* induces a strong, STING-dependent, type I IFN response, we next wished to confirm that IFN-β secretion was dependent upon STING. Indeed, treatment of *Sting*^*Gt/Gt*^ macrophages with live *S*. *aureus* resulted in a 75% decrease in IFN-β production when compared to WT macrophages (Fig 5A).

We next examined how the STING adaptor is activated by live *S*. *aureus*. STING can be activated either by di-cyclic nucleotides produced by bacteria or indirectly by the cytosolic DNA sensor cyclic GMP-AMP synthase (cGas), which produces the small molecule cGAMP to activate STING [19,38,39]. *S*. *aureus* can produce a similar di-cyclic nucleotide, di-cyclic AMP, through the dacA protein, which encodes a di-adenylate cyclase; this was recently shown to induce type I IFN in response to *S*. *aureus* biofilms [18,40]. *S*. *aureus* also possesses an Esx secretion system [41], similar to those in *L*. *monocytogenes* and *M*. *tuberculosis* that are responsible for activation of cGAS [19,20].

To clarify the mechanism by which *S*. *aureus* activates STING, we compared induction of *Ifnb1* mRNA and IFN-β1 protein by live *S*. *aureus* in *Sting*^*Gt/Gt*^ and *cGas*^-/-^ macrophages. Because all of the experiments to this point utilized the DU5938 strain of *S*. *aureus* that lacks hemolysins, we also wished to examine whether the cGAS-STING pathway activates *Ifnb1* expression in response to strains of *S*. *aureus* that are more clinically relevant. We chose a USA300 LAC strain of methicillin-resistant *S*. *aureus* (MRSA), one of the major causes of community and hospital acquired *S*. *aureus* infections in the United States, as well as *S*. *epidermidis*, a commensal staphylococcal species.

The DU5938 strain of *S*. *aureus* induced the dose-dependent activation of *Ifnb1* mRNA through cGAS-STING (Fig 5B), and induced IFN-β protein production through cGAS-STING as well, with a greater dependence on STING (Fig 5C). Infection of macrophages with USA300 strain resulted in greater induction of *Ifnb1* mRNA and IFN-β1 protein from mouse macrophages when compared to the DU5938 hemolysin-deleted strain, and this induction was similarly dependent on cGAS and STING (Fig 5B and 5C). The *S*. *epidermidis* strain led to weaker but significant induction of *Ifnb1* mRNA and IFN-β protein, with strong STING and cGAS dependence (Fig 5C). Additionally, highly purified DNA from an overnight culture of the USA300 strain of S. aureus induced *Ifnb1* mRNA expression in a dose dependent manner in WT, but not *Sting*^*Gt/Gt*^ or *cGas*^*-/-*^ macrophages (Fig 5D). These findings indicate that the cytosolic DNA sensor cGAS is the predominant sensor resulting in STING-dependent activation of type I IFN in murine macrophages in the early response to *S*. *aureus*, although residual activation through STING appears to occur either through another DNA sensor or through direct activation by di-cyclic nucleotides produced by *S*. *aureus*, as observed with Streptococcal species [22].

We next wished to determine whether the cGAS-STING pathway contributes to induction of the type I IFN response in human cells. To accomplish this, we used human THP-1 cells that had CRISPR/Cas9-mediated deletions of the *cGAS* or *STING* genes [22,42]. Similar to mouse cells, induction of *Ifnb1* mRNA was significantly diminished in both *cGas*^*-/-*^ and *Sting*^*Gt/Gt*^ cells, with a slightly stronger inhibition in the STING mutant cells (Fig 5E), confirming that human monocytic cells utilize cGAS-STING to induce type I IFN.

### Opposing roles of MyD88 and STING in cutaneous host defense to *S*. *aureus*

Since signaling through the TLR and STING pathways contributes to the majority of transcriptional response to live *S*. *aureus* in cultured macrophages, we next wished to determine the roles of these pathways in cutaneous infection. MyD88 signaling was previously shown to be critical for cutaneous host defense to *S*. *aureus* [1,2], but the role of STING in the cutaneous response to *S*. *aureus* is unclear. To test the role of these PRR pathways, we utilized a model of cutaneous infection using a bioluminescent *S*. *aureus* strain (Xen36) to track the infection over time, as bioluminescence directly correlates with viability and bacterial numbers [43] and a similar bioluminescent *S*. *aureus* was used to show the importance of MyD88 in cutaneous infection with *S*. *aureus* [3].

We first confirmed that Xen36 induced type I IFN responses through cGAS-STING *in vitro*, and found that Xen36 similarly activates early *Ifnb1* mRNA through cGAS and STING (Fig 5F). We next compared cutaneous infection of *Myd88*^-/-^ and *Sting*^*Gt/Gt*^ mice to WT mice. We chose to use *Sting*^*Gt/Gt*^ mice instead of *cGas*^*-/-*^ mice because *cGAS*^*-/-*^ mice exhibited residual *Ifnb1* induction. In our model, following injection of 1x10^6^ bacteria in the skin of WT mice, all mice developed an erythematous, indurated plaque with scale on their back by the second day post-infection. About 50% of wild type mice developed an ulceration by the 4^th^ day post-infection, likely depending on the depth of injection of the Xen36 inoculum (dermal or sub-dermal). The bioluminescence reached its maximal intensity on Day 3 following infection regardless of whether the mice developed an ulceration, and the signal then slowly lost intensity until approximately Day 10–12, when the signal was lost.

**Fig 6:**
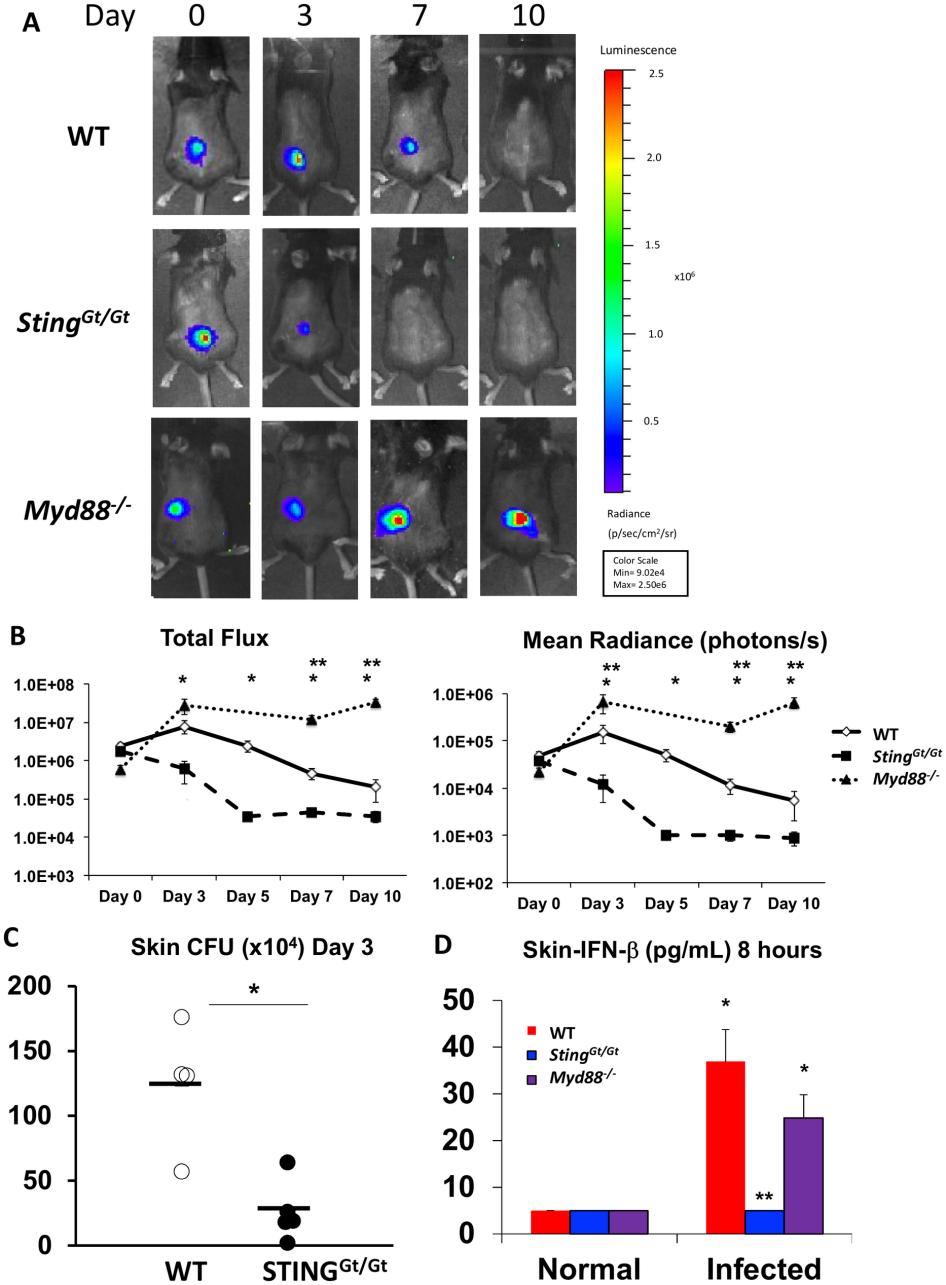
*Sting*^*Gt/Gt*^ mice display improve clearance of *S*. *aureus* following cutaneous challenge. (A) Representative WT and *Sting*^*Gt/Gt*^ bioluminescent image of one mouse of each strain following cutaneous injection of 1x10^6^ Xen36 strain of *S*. *aureus*. (B) Left panel shows Total Flux over 5 minutes, and Right panel shows Mean Radiance (Photons/second). * denotes p<0.05 by one way ANOVA with Tukey’s test for *Sting*^*Gt/Gt*^ mice when compared to WT mice at each time point and represents an n = 8 mice over two separate experiments. ** denotes p<0.05 by one-way ANOVA with Tukey’s test at each time point for *Myd88*^-/-^ mice when compared to WT mice at each time point and represents an n = 5 for *Myd88*^-/-^ compared to n = 8 WT mice. (C) Confirmation of bioluminescence data by culture of infected skin 3 days following infection confirms decreased bacterial counts in the skin of *Sting*^*Gt/Gt*^ mice (* p<0.05 by t test). (D) 8 hours after inoculation into WT and *Sting*^*Gt/Gt*^ mice, skin was harvested and IFN-β was measured by ELISA. *Sting*^*Gt/Gt*^ mice displayed an impaired ability to induce IFN-β following *S*. *aureus* infection (n = 6 WT and *Sting*^*Gt/Gt*^, n = 5 *Myd88*^*-/-*^; One way ANOVA with Tukey’s test * denotes p<0.05 compared to baseline; ** denoted p = 0.003 vs WT infected). All error bars denote standard error of the mean.

First, we confirmed that TLRs participate in cutaneous host defense to *S*. *aureus*. Unlike the WT mice, all *Myd88*^*-/-*^ mice injected with the same inoculum of *S*. *aureus*, developed progressively enlarging ulcerations that started four days after infection. MyD88^-/-^ mice displayed a greater bioluminescence signal than WT mice starting at 3 days post infection, and this signal remained elevated until mice were euthanized at 12 days post infection (Fig 6A and 6B). After confirming that *Myd88*^*-/-*^ mice displayed impaired clearance of *S*. *aureus*, we performed similar experiments in *Sting*^*Gt/Gt*^ mice. We found that *Sting*^*Gt/Gt*^ mice displayed a more rapid clearing of bioluminescent *S*. *aureus* in comparison to WT mice (Fig 6A and 6B). While lesions in about half of *Sting*^*Gt/Gt*^ mice also developed an ulceration (similar to WT mice), the *Sting*^*Gt/Gt*^ mice that ulcerated typically ulcerated earlier than WT mice, with the majority developing ulceration by two days post infection.

This finding suggests that STING signaling *in vivo* impairs cutaneous host defense against *S*. *aureus*. Whereas many WT mice still exhibited a bioluminescent *S*. *aureus* signal in their wound at 7 and 10 days post-infection, the *Sting*^*Gt/Gt*^ mice displayed significantly improved clearance at Day 3 and Day 5, and complete clearance of bioluminescent signal at Day 7. Standard bacteriological plating of infected skin from skin harvested on Day 3 following *S*. *aureus* infection confirmed that *Sting*^*Gt/Gt*^ mice possessed decreased bacterial burdens when compared to WT mice (Fig 6C).

### TLR signaling promotes while STING signaling impairs local IL-1β production and neutrophil recruitment

To determine whether STING signaling contributes to early IFN-β production *in vivo*, we measured IFN-β protein in the skin 8 hours after infection. While WT mice were capable of inducing IFN-β in the skin, *Sting*^*Gt/Gt*^ mice had a significantly reduced ability to produce IFN-β (Fig 6D). *Myd88*^*-/-*^ mice did not display a significant difference in early IFN-β production in the skin in comparison to WT mice. These data suggest that STING activation contributes to IFN-β production and diminishes the ability to clear *S*. *aureus*.

**Fig 7:**
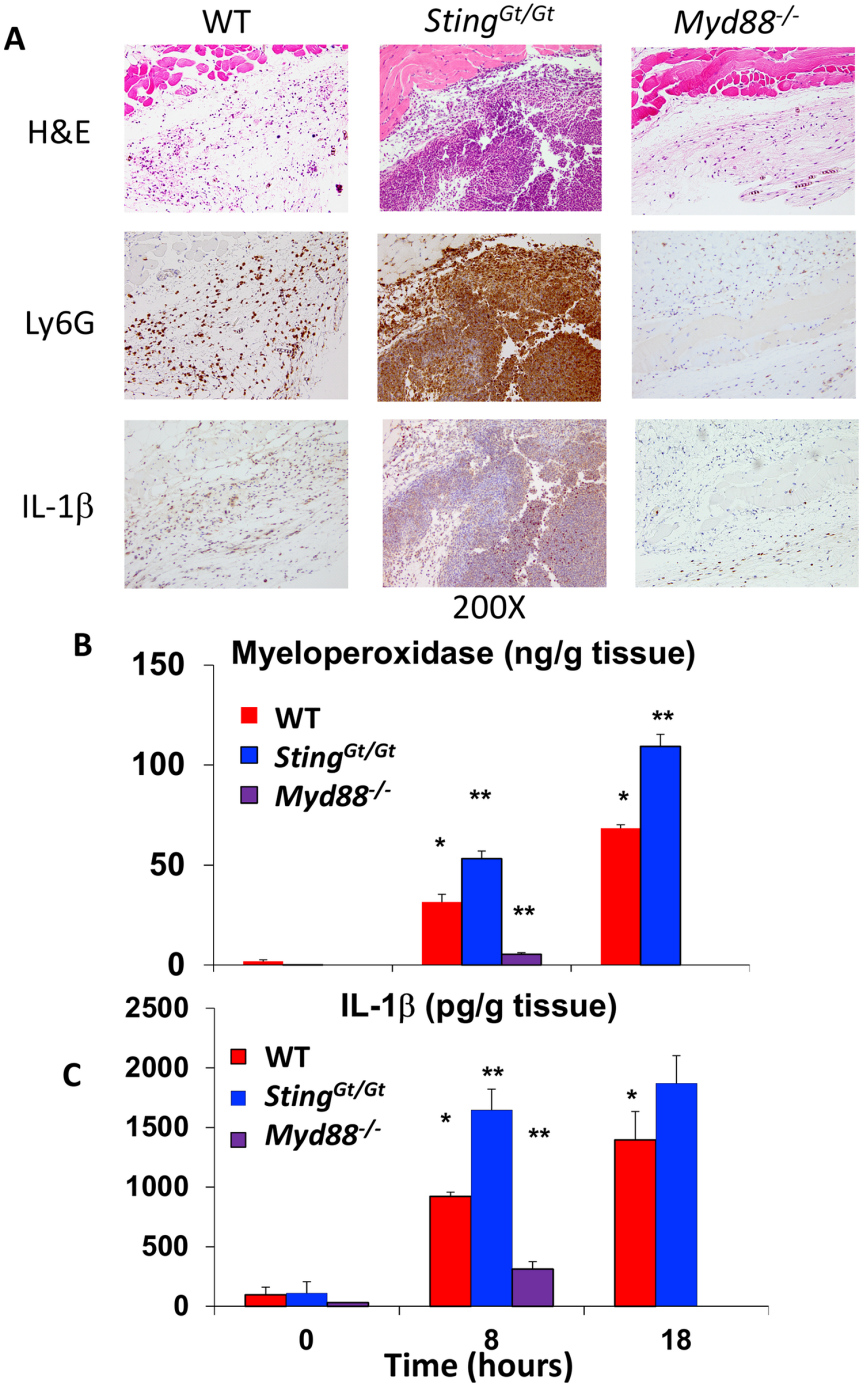
STING signaling suppresses neutrophil recruitment and IL-1β production following cutaneous infection with *S*. *aureus*. (A) 18 hours after infection, skin was harvested and histology and immunohistochemistry was performed for Ly6G and IL-1β. Representative example of high power (200X) images of skin from a WT, *Sting*^*Gt/Gt*^ and *Myd88*^-/-^ mouse are shown. (B) Skin homogenates from WT and *Sting*^*Gt/Gt*^ mice at 8 and 18 hours (n = 6 per group) and *Myd88*^*-/-*^ mice at 8 hours (n = 5) * p<0.05 vs baseline; ** p<0.05 vs WT stimulated at same time point sample by one way ANOVA with Tukey test. (C) IL-1β expression at 8 and 18 hours after *S*. *aureus* infection in WT (n = 6 per time point), *Sting*^*Gt/Gt*^ mice (n = 6 per time point), and *Myd88*^-/-^ mice (n = 5, 8 hour time point only). * p<0.05 vs baseline; ** p<0.05 vs WT stimulated at same time point by one way ANOVA with Tukey test. All error bars denote standard error of the mean.

To further understand the mechanism by which STING activation influences cutaneous host defense to *S*. *aureus*, we first examined tissue histologically at 18 hours (prior to any ulceration) following infection. Although the infiltration of polymorphonuclear cells was present diffusely in the dermis and panniculus in the skin of WT mice, the presence of a well-defined abscess was only rarely seen (1/8) (Fig 7A). However, in *Sting*^*Gt/Gt*^ mice, the majority of the samples already possessed a well-defined abscess (5/8) with greater numbers of polymorphonuclear cells in the well-organized collection. *Myd88*^*-/-*^ mice displayed small numbers of neutrophils in the panniculus, but no mice (0/5) displayed a well-defined abscess.

Immunohistochemical staining for Ly6G confirmed the presence of neutrophils in the WT skin, with lower numbers in *Myd88*^*-/-*^ skin, as well as the well-formed collection of neutrophils in the *Sting*^*Gt/Gt*^ skin (Fig 7A). To quantify neutrophil infiltration into tissue, we measured myeloperoxidase, a marker of neutrophil activity following *S*. *aureus* infection. Myeloperoxidase was increased in skin of *Sting*^*Gt/Gt*^ mice compared to WT mice at 8 and 18 hours following infection (Fig 7B). On the other hand, there was a large impairment in the ability of *Myd88*^*-/-*^ mice to recruit neutrophils to the skin, as they demonstrated an approximately 70% reduction in myeloperoxidase levels 8 hours after infection (Fig 7B). These data demonstrate that *S*. *aureus* activation of STING impairs while MyD88 activation is required for neutrophil recruitment in the skin.

IL-1β is known to be critical for neutrophil recruitment in the skin in response to *S*. *aureus*, and type I IFN can inhibit inflammasome-mediated induction of IL-1β [1, 12]. Furthermore, prior studies of group A Streptococcus revealed a role for type I IFN in regulating IL-1β expression from the skin [15]. We therefore investigated whether *S*. *aureus* activation of STING impairs local IL-1β production in the skin.

Immunohistochemical analysis revealed that IL-1β was produced in the skin of WT mice infected with *S*. *aureus* at 18 hours; the majority of the IL-1β was produced by recruited neutrophils (Fig 7A). There was stronger immunohistochemical staining in neutrophils of *Sting*^*Gt/Gt*^ mice and very low levels of IL-1β staining in neutrophils of *Myd88*^*-/-*^ mice (Fig 7A).

We next measured IL-1β in skin homogenates to quantify whether IL-1β levels are higher in *Sting*^*Gt/Gt*^ mice. Indeed, we found that IL-1β was increased at 8 and 18 hours following *S*. *aureus* infection in WT mice, and *Sting*^*Gt/Gt*^ mice demonstrated a significant increase in IL-1β expression (Fig 7C). *Myd88*^*-/-*^ mice demonstrate a 60% reduction in IL-1β at 8 hours post-infection in comparison to WT mice, confirming the essential role of MyD88 for IL-1β induction (Fig 7C). Taken together, our findings demonstrate that although TLR-dependent activation of MyD88 signaling induces protective immunity to limit *S*. *aureus* infection, *S*. *aureus* utilizes STING signaling to subvert cutaneous host defense.

## Discussion

Studies of the innate immune response to infection by *S*. *aureus* and other bacterial pathogens have typically identified several PRR pathways capable of sensing the pathogen and contributing to host defense. Although multiple PRR pathways can play a role in host defense to a given pathogen, the relative contribution of each for the gene expression response elicited by infection generally has not been characterized. An understanding of relative contributions requires a quantitative analysis of the response in WT cells, as well as the response that remains after each PRR pathway is eliminated. This general approach has limitations, in that multiple pathways may each make small contributions to the activation of some genes; other genes may be activated by two or more pathways in a redundant fashion. However, the approach allows the identification of dominant PRR regulators of many genes and represents an essential first step toward a full understanding of the host-pathogen interactions that regulate the immune response, as well as evasion of the response.

In this study, we carried out an initial, quantitative analysis of the gene expression response in mouse BMDMs to infection by live *S*. *aureus*, and a comparison of the BMDM response to the live and HK pathogen. We focused on the most strongly induced genes because of the difficulty interpreting loss-of-function results when examining weakly induced genes. For example, if a gene is induced by only 2.5-fold by the WT pathogen, it is difficult to interpret the importance of a PRR pathway when elimination of that pathway reduces induction to 1.7-fold or 2.1-fold. This issue remains a challenge even when analyzing strongly induced genes. However, by focusing on genes that are induced by at least 5-fold, it is possible to have greater confidence in the contribution of a PRR pathway when a strong quantitative effect is observed in a loss-of-function experiment.

For these loss-of-function experiments, we arbitrarily chose to define a gene as “dependent” on a particular signaling pathway if its expression level in the mutant cells was reduced to <50% of the expression level observed in WT cells. We chose to emphasize these relatively strong effects to increase confidence in the results and also to identify the dominant regulators of each gene. The pathways studied clearly make smaller contributions to the induction of additional genes, and some of those contributions reach statistical significance. Furthermore, we would not be surprised to find modest contributions of pathways that were not examined in this study. Nevertheless, our finding that the vast majority of the strongly induced genes exhibit expression levels that are diminished to <50% of WT in either *Myd88*^*-/-*^*Trif*^*-/-*^ or *Sting*^*Gt/Gt*^ BMDMs highlights the dominant roles of these two pathways in the early transcriptional response to *S*. *aureus*.

One notable feature of our study is that we used a strain of *S*. *aureus* deficient in the pore-forming toxins (α/β/γ hemolysin- and Panton-Valentine leucocidin toxin). The parental strain (8325–4) from which DU5938 was derived also lacks phenol soluble modulin α 3 (PSM-α3), another virulence factor that can cause cytotoxicity in macrophages, and the DU5938 strain is, therefore, likely to also lack PSM-α3 [7]. This strain was chosen because macrophages in a cell culture system do not have the benefit of complement, antimicrobial peptides, or natural antibodies that can effectively neutralize *S*. *aureus in vivo*, and the use of toxin-sufficient strains rapidly killed macrophages, with significant macrophage death starting as early as 2 hours. In response to the WT strain, this time point corresponded to an acute burst of transcriptional activity, but by 4 hours, many (8 of 10) genes tested had decreased to within 25–50% of basal activity, likely because considerable cell stress or death was occurring. Thus, we wished to evaluate the mechanisms responsible for early gene induction by the live and HK pathogen in the absence of the effects of apoptosis/necroptosis/necrosis that these pore-forming toxins can cause, which include the non-specific release of danger associated molecular patterns (DAMPs) from the dying cells. We acknowledge that the pore-forming toxins can contribute to gene expression by activating inflammasome pathways. However, the inflammasome generally requires more than 4 hours to be activated, as activation of caspase-1, transcription and translation of pro-IL-1β, and proteolytic cleavage to mature IL-1β, are all required before the inflammasome can activate further transcription through IL-1R and MyD88.

Although many innate immune sensors are undoubtedly activated by *S*. *aureus*, our results revealed that two pathways, the TLR and DNA-cGAS-STING pathways, represent dominant regulators of early transcription of the majority of genes that are strongly induced by live *S*. *aureus* in cultured mouse BMDMs; a hypoxia response appears to account for the induction of many of the small number of early genes induced in a TLR/STING-independent manner. Although the gene expression responses to live and HK *S*. *aureus* are very similar at first glance, many of the genes that exhibit STING-dependence in response to the live pathogen are activated by TLR pathways in response to the HK pathogen. These results are consistent with the fact that HK *S*. *aureus* remains in the phagolysosome, while live *S*. *aureus* is capable of either escaping the phagolysosome and entering the cytosol or secreting substances into the cytosol that can activate cytosolic PRRs, including cGAS, STING, or NOD receptors [6,7] Furthermore, the hypoxia response elicited by the live pathogen was not observed with the HK pathogen. An analysis of mutant mouse strains demonstrated that the STING pathway serves as a negative regulator of host defense to *S*. *aureus* infection, with the STING pathway promoting a type I IFN response and negatively regulating IL-1β expression and neutrophil recruitment.

Our identification of a TLR/STING-independent hypoxia program in macrophages exposed to live bacteria was also of special interest. Hypoxia has been found to serve as a metabolic switch to “train” macrophages to become more antimicrobial; it has been detected in response to *S*. *aureus* infections *in vitro* and *in vivo*, and may be related to a decrease in oxygen tension in infected tissue/cells [32,44]. While TLR signaling alone can induce a hypoxia program, we found an additional hypoxia signature in the absence of TLR signaling. Recently, oxygen consumption and glycolysis by *S*. *aureus* was shown to contribute to a hypoxia response by activating HIF-1α [45]. This HIF-1α activation, in turn, augmented IL-1β production and promoted keratinocyte and macrophage activation, while blocking glycolysis worsened cutaneous infections. Acute and chronic hypoxia can alter host defense responses to *S*. *aureus* and triggering the hypoxia machinery has been suggested as a potential therapeutic target [46].

The cytosolic DNA pathway can be activated by multiple viruses, bacteria, and mycobacterial species upon access of the pathogen or its DNA to the cytosol [20,47]. *Streptococcus pneumoniae* and group B streptococcus, both of which are Gram-positive bacteria similar to *S*. *aureus*, were recently shown to activate type I IFN responses in macrophages through this same pathway[22,48]. Other PRRs, including TLR2, TLR7, TLR9, and NOD2, were shown to be involved in type I IFN induction in response to *S*. *aureus* in other cell types using different treatment conditions and lengths of time in culture [4,5,16,17]. Importantly, we focused on early time points to less ambiguously determine the pathways involved in macrophage activation and found that the activation of STING requires the presence of living bacteria, and can be induced by both less virulent *S*. *epidermidis* and *S*. *aureus* species along with more virulent USA300 strains of MRSA. *S*. *aureus* used the cytosolic DNA sensor cGAS to activate STING, but it can also activate STING in a cGAS-independent manner, demonstrating similar redundant activation of the pathway to group B streptococcus [22]. The fact that the USA300 strain resulted in higher IFN-β induction through cGAS-STING is likely due to the increased cell death associated with this strain, as release of host DNA from dying cells likely contributes to the increased IFN-β production, since basal and stimulated type I IFN is regulated by cell death [49]. This is corroborated by the fact that *S*. *epidermidis*, which did not cause considerable cell death, also induced lower levels of IFN-β than either DU5938 or USA300.

The role of type I IFN in response to bacterial pathogens, especially extracellular bacteria, is complex [50,51]. An inability to induce type I IFN was shown to be detrimental in *S*. *aureus* skin infection and adding IFN-β resulted in improved clearance of *S*. *aureus* [29]; however, in a pneumonia model, type I IFN was shown to be detrimental [4,11,17]. Type I IFN can decrease neutrophil chemotaxis and limit neutrophil infiltration, and it leads to increased susceptibility to *S*. *aureus* and *Streptococcus pneumonia* following influenza infection [52,53]. A strain of *S*. *aureus* that elicits high levels of type I IFN induction also displays increased virulence in a pneumonia model [11,47,53]. Molecularly, type I IFNs can directly inhibit the transcription of IL-1β as well as the processing of pro IL-1β protein into mature IL-1β through the inflammasome [12–14]. This inhibition of IL-1β processing by type I IFN has been shown to limit host defense to *M*. *tuberculosis*, *Candida albicans*, and Group A *Streptococcus*, and is likely the mechanism by which STING-dependent type I IFN is limiting IL-1β production in response to *S*. *aureus*. Understanding how *S*. *aureus* triggers immune pathways and the relative contributions of known and/or novel PRR pathways to *S*. *aureus*-initiated transcriptional cascades may lead to novel strategies to combat infection. We are the first to identify the activation of cGAS by *S*. *aureus* DNA through the STING adaptor as a major contributor to the initial macrophage type I IFN response to live *S*. *aureus*.

For our studies, we implemented a model of cutaneous infection by *S*. *aureus* to determine the role of the STING pathway in local host defense. We wished to avoid the systemic inflammatory response that a large inoculum of bacteria would cause, since systemic inflammation and bacteremia can compromise local host defense and make interpretation of results more difficult. Using our model, we found that *S*. *aureus* activates STING in the skin, which results in the elaboration of IFN-β. Activation of this STING-dependent IFN-β production results in impaired local clearance of *S*. *aureus*. Since IL-1β is critical for neutrophil recruitment to *S*. *aureus* infection sites [3,54], the loss of early type I IFN in STING^*Gt/Gt*^ mice likely accounts for the increase in IL-1β and enhanced neutrophil recruitment at the site of infection.

The two major early pathways activated by *S*. *aureus* within hours of infection in macrophages led to diametrically opposed outcomes following *in vivo* infection with *S*. *aureus*. While both pathways synergized to induce the transcription of some genes in macrophages, the two pathways had opposite effects on host defense *in vivo*. This likely signifies that the effects of STING signaling on downstream transcriptional cascades are uncoupled from the effects of IFN-β produced *in vivo*. During cutaneous infection, the TLR-dependent pathway is critical for early local host defense while activation of the cGAS-STING pathway results in immune evasion by *S*. *aureus*.

Recently, Castiglia et al. showed that during an overwhelming cutaneous *S*. *pyogenes* infection that resulted in bacterial dissemination, activation of type I IFN systemically suppressed IL-1β driven inflammation, limiting organ damage [15]. These results were consistent with previous findings that IFN-β produced during influenza infection can diminish IL-1β and host immunity to *S*. *aureus* [53,55]. Furthermore, activation of the cytosolic DNA pathway through STING in response to the intracellular pathogens, *Listeria monocytogenes* and *M*. *tuberculosis*, was shown to similarly limit host defense to infection [39,56].

Taken together, these studies suggest that TLR- and inflammasome-dependent IL-1β production is required for host defense to bacterial infection, but may become detrimental in the case of overwhelming infections. Thus, type I IFN, including that produced through the cGAS-STING pathway, can inhibit IL-1β processing, providing a rheostat on IL-1β production. During local infections, this type I IFN can impair host defense, whereas in severe infections, this type I IFN can diminish systemic inflammation caused by overproduction of IL-1β. Hence, targeting cGAS and/or STING may remove detrimental type I IFN without affecting protective innate immune host defense pathways. Another interpretation is that part of the gene program that requires STING and type I IFN includes genes that are critical in triggering T cell activation and adaptive immunity (Fig 3D). Perhaps the host is activating the STING pathway, a pathway that may lead to diminished initial host defense, in order to trigger activation of adaptive immunity to confer longer protection against a rechallenge.

## Materials and methods

### Ethics statement

The mouse studies described in this manuscript were performed under the written approval of the UCLA Animal Research Committee (ARC) in accordance to all federal, state, and local guidelines. All studies were carried out under strict accordance to the guidelines in The Guide for the Care and Use of Laboratory Animals of the National Institutes of Health and the accreditation and guidelines of the Association for the Assessment and Accreditation of Laboratory Animal Care (AALAC). The protocol/permit/project license number assigned by the IACUC/ethics committee that approved under UCLA ARC Protocol Number 2015-021-01. *S*. *aureus* infections were performed under isofluorane anesthesia and all efforts were made to minimize animal pain and discomfort.

### Mice

All mice used have a C57BL/6J genetic background, *Myd88*^*-/-*^ mice, *Sting*^*Gt/Gt*^ mice, and *cGas*^*-/-*^ mice were purchased from Jackson Laboratory (Bar Harbor, ME). *Myd88/Trif*^*-/-*^ mice were a kind gift of Greg Barton[57].

### Human and mouse and cell culture

Human wild type THP-1 cells or those deleted of *cGAS* and *STING* were a kind gift of V. Hornung and were cultured in RPMI-1640 (Gibco Laboratories; Gaithersburg, MD) with 10% FBS (Omega Scientific; Tarzana, CA) and 0.05 mM 2-mercaptoethanol (Sigma-Aldrich; St. Louis) [42]. Cells were grown to ~80% confluence and treated with 10 ng/ml phorbol myristate acetate (Sigma) for 2 hours prior to stimulation.

BMDMs were isolated and differentiated from WT or mutant/deficient mice as previously described in [28]. Briefly, bone marrow isolated from femurs and tibias from male mice at 6–10 weeks of age were cultured in BMDM media consisting of DMEM (Gibco) with 20% FBS (Omega), CMG conditioned media containing M-CSF (cell line was a kind gift from G. Cheng) [58], and 1X Pen/Strep (Gibco) for 6 days. All media was washed twice with PBS (Corning), then antibiotic-free BMDM media was added prior to addition of bacteria. CMG was prepared from L929 cells.

### Bacterial strains and culture

Strain DU5938, a mutant of the WT 8325–4 lab strain triple defective mutant in α, β, and γ hemolysins, was a kind gift of T. Foster [26]. In other experiments, the USA300 LAC strain (JE2; ATCC/BEI Resources; Manassas, VA) was used. For *S*. *epidermidis*, the Winslow and Winslow strain (ATCC) was used. The Xen36 strain (bioluminescent strain of 8325–4 lab strain) was purchased from Perkin Elmer (Walthum, MA). Staphylococcal bacteria were inoculated from single colonies and grown shaking at 200 rpm overnight in LB (Fisher Scientific) at 37°C. The following morning, a 1:40 dilution of the overnight culture was shaken in LB for 3 hours, washed twice with PBS (Corning Life Sciences; Manassas, VA), and resuspended. Bacterial concentrations were estimated with a spectrophotometer (Beckman DU 640B Spectrophotometer, Beckman Coulter, Inc., Fullerton, CA) by determining the absorbance at 600 nm (A600). Colony-forming units (CFUs) were verified by plating dilutions of the inoculum onto LB agar overnight. HK *S*. *aureus* was obtained by placing live *S*. *aureus* culture in a 65°C water bath for 1 hour[26]. To obtain highly purified DNA from *S*. *aureus*, DNA was extracted from an overnight culture using the DNAZol Reagent (ThermoFisher; Canoga Park, CA) per manufacturer recommendations. Following extraction, DNA was treated with 1μg/ml of RNase A. Then the DNA was purified by phenol chloroform extraction. The A_260_/A_280_ ratio of the purified DNA that was used in the experiments herein was 1.81.

### *In vitro* infections and stimulations

Mouse macrophages were stimulated on day 6 following differentiation. Human and mouse macrophages were infected with bacterial strains at 10 M.O.I. in antibiotic free MDM and BMDM media. Macrophages were infected with *S*. *aureus* for 0, 30, 60, 120, and 240 minutes at 37°C or treated with HK *S*. *aureus* for the same time points. Media was obtained at the 240-minute time point for cytokine determination by ELISA.

### qPCR

RNA was isolated and reverse transcribed as described[58]. Briefly, RNA was extracted using TRI-reagent (Molecular Research Center; Cincinnati, OH), treated with RNase-free DNaseI, and purified using an RNeasy kit (Qiagen Inc; Valencia, CA). Quantified RNA (2 μg) was reverse-transcribed using Omniscript RT Kit (Qiagen) and random hexamer primers. cDNA fragments were analyzed by qPCR using SensiMix Plus (Bioline; Taunton, MA) and the iCycler System (Bio-Rad Laboratories; Irvine, CA) or a 7900HT (Applied Biosystems). PCR amplification conditions were 95°C (3 min) and 45 cycles of 95°C (15 sec), 60°C (30 sec), and 72°C (30 sec). Primer pairs for mouse and human IFN-β, respectively, are as described [59,60]. Results for IFN-β were compared to β-actin or h36b4 (Thermo-Fisher), which were used as housekeeping internal controls for mouse or human, respectively.

### RNA-seq

RNA was isolated as described[27]. Strand-specific libraries were generated from 400 ng total RNA using the TruSeq RNA Sample Preparation Kit v2 (Illumina), with modifications using the “dUTP” method [61]. cDNA libraries were single-end sequenced with a length of 50bp on an Illumina HiSeq 2000.

All bioinformatics analyses were conducted using Galaxy[62]. Reads were aligned to the mouse genome (NCBI37/mm9) with TopHat v1.3.3 and allowed a maximum of one alignment with up to two mismatches per read. mRNA RPKM values were calculated using Seqmonk (http://www.bioinformatics.babraham.ac.uk/projects/seqmonk/). RPKMs were calculated by dividing mapped exonic reads by the length of the mature mRNA product.

All RPKMs represent an average from at least two biological replicates. A gene was included in the analysis if it met all of the following criteria: The maximum RPKM reached 1 at any time point, the gene was induced at least 5-fold, and the induced expression was significantly different from the basal (*P*<0.05) as determined by the DESeq package in R Bioconductor[63]. P-values were adjusted using the Benjamini-Hochberg procedure for multiple hypothesis testing[64]. The splice variant with the largest RPKM was included in the analysis.

To normalize the data, the basal RPKM in WT samples was set at 0% and the maximum WT RPKM at 100% for each gene. In the mutant strains, percent expression was calculated using this scale substituting only the maximum WT RPKM with the maximum mutant RPKM.

For promoter enrichment, PScan software (http://159.149.160.88/pscan) using Jaspar 2016 motif analysis examining -450 to +50 base pairs of the promoter of groups of interest. For Gene Ontogeny analysis, gene families were inputted into ENRICHR software[31,65]. The GO Biologic Process function was used for gene ontogeny classification. The top 5 significant GO terms were used. Both the lighter color and longer length of the bars denote stronger significance of the identified pathway.

### Mouse model of cutaneous *S*. *aureus* infection

All procedures were approved by UCLA Animal Research Committee. The mice were shaved on the back and inoculated subcutaneously with 100 μl of mid logarithmic growth phase *S*. *aureus* strain Xen36 (∼1 × 10^6^ CFUs/100 μl = 1:10 dilution of A600 of 0.5/ml) in sterile pharmacy grade saline (0.9%) by a 27-gauge needle and a tuberculin syringe (Abbott Laboratories; Chicago, IL). In one experiment, a subset of WT and Sting^Gt/Gt^ mice (n = 4–5 mice) was used to confirm bioluminescence results by determining CFU from skin on Day 3.

### Quantification of in vivo *S*. *aureus*

In vivo bioluminescence was performed with the Xenogen IVIS imaging system (Xenogen Corporation; Alameda, CA) at the Crump Institute for Molecular Imaging at UCLA as previously described[66]. Mice were anesthetized via isofluorane injection. Data are presented on color scale overlaid on a gray-scale photograph of mice and quantified as total flux and average radiance (photons/s) within a circular region of interest (1 × 10^3^ pixels) with Living Image software (Xenogen) (lower limit of detection: 1 × 10^4^ photons/s).

### Tissue embedding and staining

For histological analysis, lesional 8 mm punch biopsy (Acuderm; Ft. Lauderdale, FL) specimens were bisected and fixed in formalin (10%) and embedded in paraffin. Hematoxylin and eosin (H&E) and immunoperoxidase labeling was performed on paraffin sections (4 μm) by the Tissue Procurement & Histology Core Laboratory and by the Histopathology Laboratory at UCLA, according to guidelines for clinical samples.

### Immunoperoxidase labeling

Detection of Gr-1 (Ly-6G)-positive cells and IL-1β expression on paraffin embedded specimens of lesional skin punch biopsy specimens were performed with a biotinylated rat anti-mouse Ly6G mAb (clone 1A8) (1 μg/ml) (BD Pharmingen, San Diego, CA) or rat anti-mouse IL-1β mAb (clone H153) (1 mg/ml) (Santa Cruz Biotechnology; Dallas, TX) by the immunoperoxidase method.

### Myeloperoxidase activity assay

Myeloperoxidase activity was used to assess neutrophil accumulation in the skin tissue using a previously reported method[67]. Briefly, 8mm punch biopsy samples were weighed and homogenized on ice in 0.01 mol/L KH2PO4 at a ratio of 1 volume tissue to 15 volumes of buffer. After centrifugation at 10,000g for 20 min at 4°, the pellets were resuspended by sonication in cetyltrimethylammonium bromide buffer (13.7 mM cetyltrimethylammonium bromide (Sigma), 50 mM KH2PO4 (Sigma), 50 mmol/L acetic acid; pH 6.0 (Sigma) at a ratio of 1 to 5 weight to volume. The supernatant was kept for ELISA analysis (see previous text). The suspension was centrifuged again at 10,000g for 15 min, and the pellet was discarded. The supernatant was then incubated in a 60°C water bath for 2 h. Myeloperoxidase activity of the supernatant was measured by the H_2_O_2_-dependent oxidation of tetramethylbenzidine. Absorbance was determined at 650 nm and compared with a linear standard curve of recombinant mouse MPO (R&D Systems, Minneapolis, MN).

### IFN-β ELISA and cytometric bead array

For detection of cytokines from skin, supernatants from the first step of the MPO assay were used. For the detection of cytokines from cell culture, supernatants from cultured macrophages were used. For the detection of IFN-β, the Mouse IFN-β Verikine ELISA kit (PBL Assay Science, Piscataway, NJ) was used according to manufacturer instructions. For detection of IL-1β, a Cytometric Bead Assay (BD Biosciences, Piscataway, NJ) was performed per manufacturer instructions.

## Supporting information

S1 Fig Most genes below induction threshold by one stimulus miss fold change criteria but are induced by other stimulus. A) RPKM and B) Fold change scatterplots of individual genes expressed following stimulation with live and/or HK *S*. *aureus*. Of note, scatterplots do not display all genes, and are limited to the ones expressed below 8 RPKM (in A) and 10-fold induced (in B) to highlight the weakly induced genes.
(TIF)

S2 Fig Integrated framework of transcriptional activation of genes induced in BMDMs by heat killed *S*. *aureus*. (A) Heat map of percentile induction of genes induced by treatment of BMDMs with HK S. aureus (equivalent of MOI 10) in WT (B6), *Myd88*^*-/-*^*Trif*^*-/-*^, or *Sting*^*Gt/Gt*^ mice reveals three distinct clusters of genes. Genes are separated into 3 clusters (I, II, and III) by mode of induction. (B) Venn diagram demonstrating the breakdown of genes in the four clusters of genes based on >50% dependence on the two main pathways induced. Of note, 358 of 364 genes induced (1 RPKM, 5 fold) are induced through TLR signaling. (C) Enriched Jaspar 2016 motifs within promoters of genes within the 3 clusters of genes defined by dependence on TLR and/or STING pathways.
(TIF)

S3 Fig Extended statistics and scores for GO: Biologic process analyses. (A) Statistical output of Enrichr GO: Biologic Process 2015 shown as a graphical representation in Fig 3D. (B) Statistical output of Enrichr GO: Biologic Process 2015 shown as a graphical representation in Fig 4B.
(TIF)

S1 Table 369 genes induced by live *S*. *aureus*. Shown is the average RPKM of 369 genes that are induced significantly by live *S*. *aureus* (average of at least 4 experiments per time point) at least 5-fold that reach a minimum 1 RPKM at least in one time point (p<0.05 by DEseq), their values in response to HK *S*. *aureus* (average of at least 2 experiments per time point), and the % inhibition in *Myd88*^*-/-*^*Trif*^*-/-*^ (DKO) macrophages or *Sting*^*Gt/Gt*^ (KO) macrophages (n = 2 experiments for each).
(XLSX)

S2 Table 364 genes induced by heat killed *S*. *aureus*. Shown is the average RPKM of 364 genes that are induced by heat killed *S*. *aureus* (average of 2 experiments) at least 5-fold that reach a minimum 1 RPKM at least in one time point (p<0.05 by DESeq), their values in response to live *S*. *aureus* (average of at least 4 experiments per time point), and the % inhibition in *Myd88*^*-/-*^*Trif*^*-/-*^ (DKO) macrophages or *Sting*^*Gt/Gt*^ (KO) macrophages (n = 2 experiments for each).
(XLSX)
