# Supplementary figures and images for: Opposing roles of Toll-like receptor and cytosolic DNA-STING signaling pathways for Staphylococcus aureus cutaneous host defense
Source: PLoS Pathog. 2017 Jul 13;13(7):e1006496. doi: 10.1371/journal.ppat.1006496 (PMC5526579; doi:10.1371/journal.ppat.1006496)

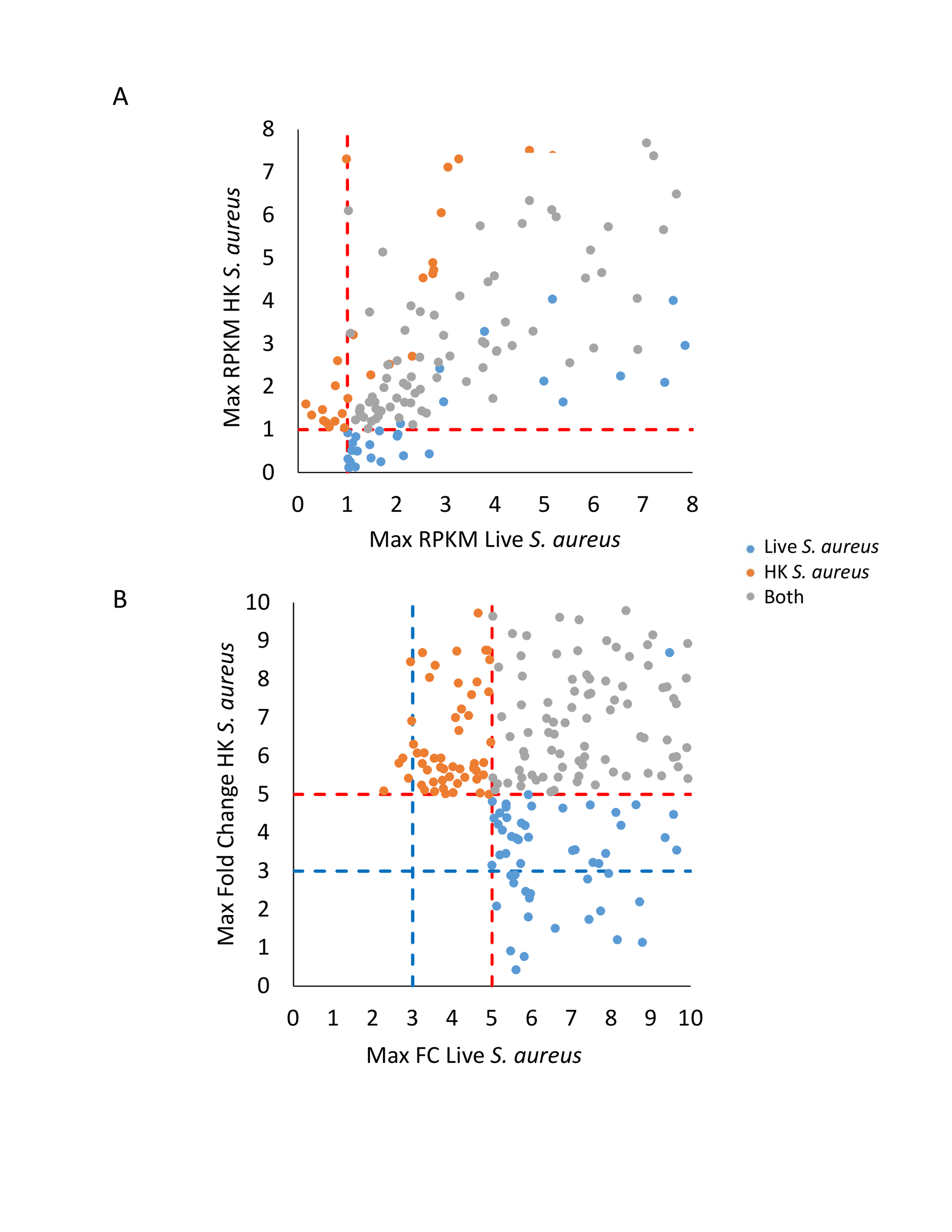

Supplement: S1 Fig — A) RPKM and B) Fold change scatterplots of individual genes expressed following stimulation with live and/or HK S. aureus. Of note, scatterplots do not display all genes, and are limited to the ones expressed below 8 RPKM (in A) and 10-fold induced (in B) to highlight the weakly induced genes. (TIF) [file ppat.1006496.s001.tif]

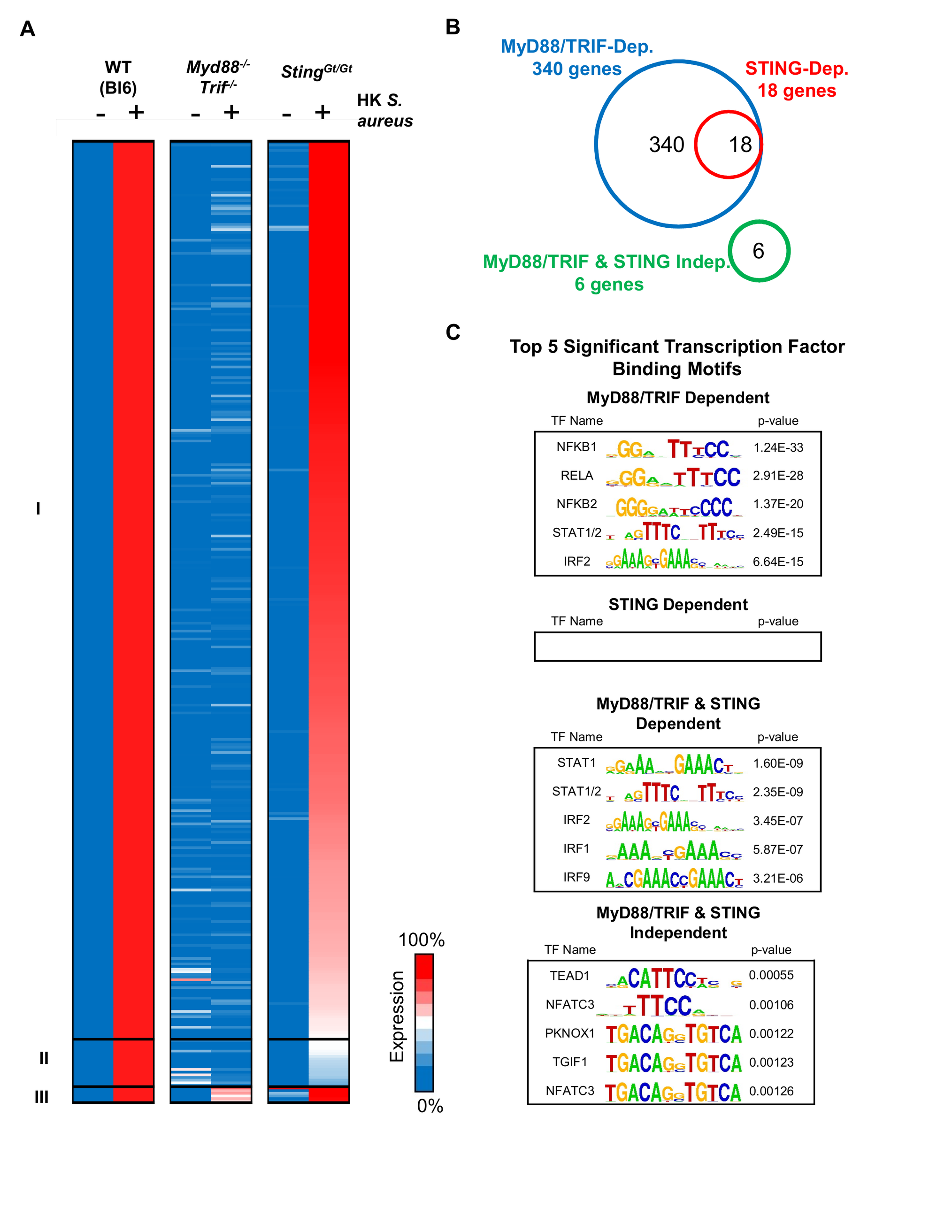

Supplement: S2 Fig — (A) Heat map of percentile induction of genes induced by treatment of BMDMs with HK S. aureus (equivalent of MOI 10) in WT (B6), Myd88-/-Trif-/-, or StingGt/Gt mice reveals three distinct clusters of genes. Genes are separated into 3 clusters (I, II, and III) by mode of induction. (B) Venn diagram demonstrating the breakdown of genes in the four clusters of genes based on >50% dependence on the two main pathways induced. Of note, 358 of 364 genes induced (1 RPKM, 5 fold) are induced through TLR signaling. (C) Enriched Jaspar 2016 motifs within promoters of genes within the 3 clusters of genes defined by dependence on TLR and/or STING pathways. (TIF) [file ppat.1006496.s002.tif]

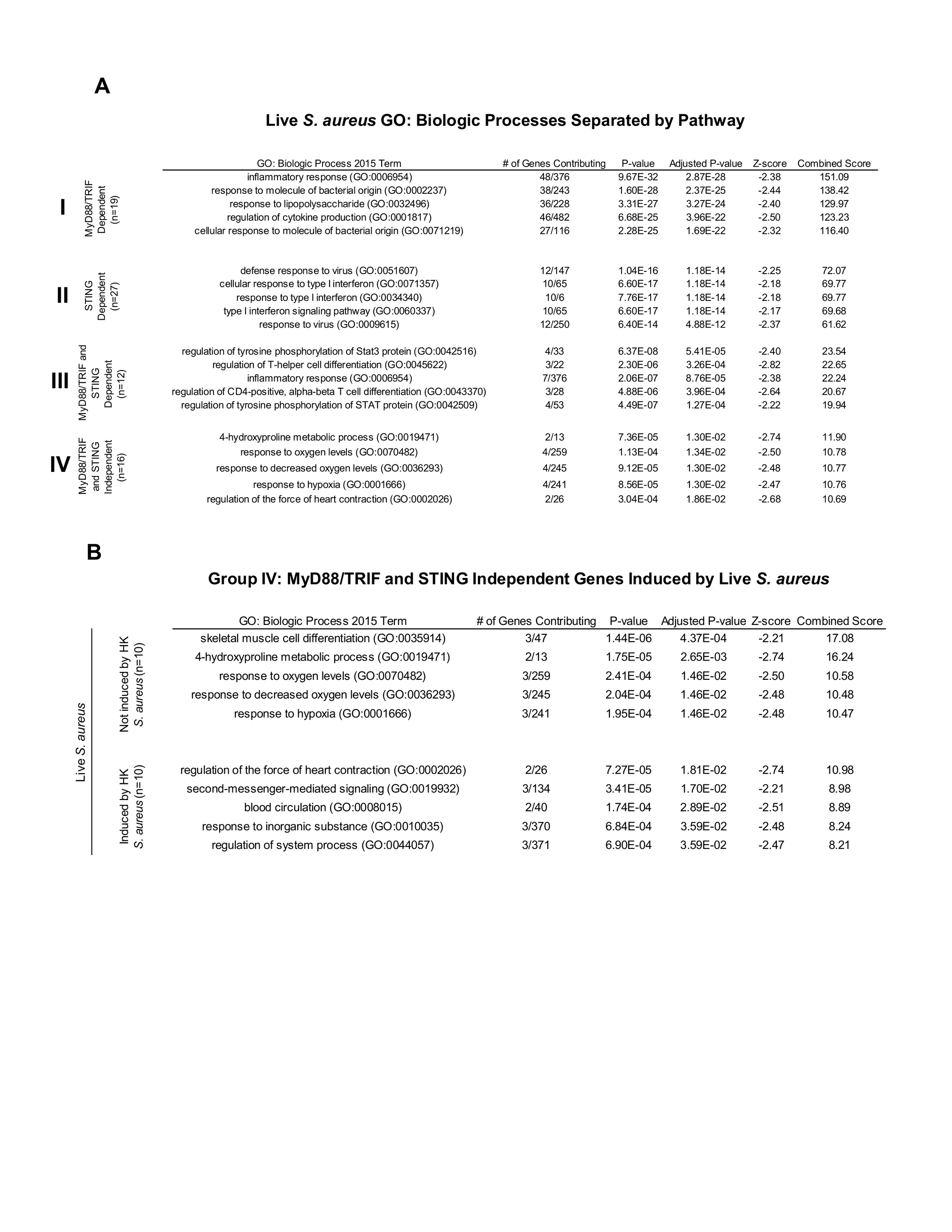

Supplement: S3 Fig — (A) Statistical output of Enrichr GO: Biologic Process 2015 shown as a graphical representation in Fig 3D. (B) Statistical output of Enrichr GO: Biologic Process 2015 shown as a graphical representation in Fig 4B. (TIF) [file ppat.1006496.s003.tif]
